# Mechanism Insight into Direct Amidation Catalyzed by Zr Salts: Evidence of Zr Oxo Clusters as Active Species

**DOI:** 10.1021/acs.inorgchem.4c02526

**Published:** 2024-10-18

**Authors:** Yujie Zhang, Jordi Puiggalí-Jou, Angelo Mullaliu, Albert Solé-Daura, Jorge J. Carbó, Tatjana N. Parac-Vogt, Francisco de Azambuja

**Affiliations:** † Department of Chemistry, KU Leuven, Celestijnenlaan 200F, 3001 Leuven, Belgium; ‡ Department de Química Física i Inorgànica, Universitat Rovira i Virgili, Tarragona 43007, Spain

## Abstract

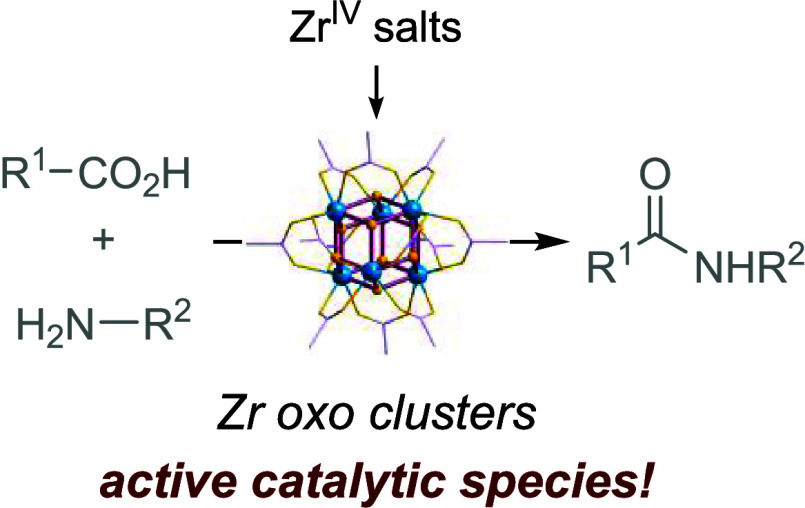

The capricious reactivity and speciation of earth-abundant metals (EAM) hinder the mechanistic understanding essential to boost their efficiency and versatility in catalysis. Moreover, metal’s solution chemistry and reactivity are conventionally controlled using organic ligands, while their fundamental chemistry in operando conditions is often overlooked. However, in this study, we showcase how a better understanding of in operando conditions may result in improved catalytic reactions. By assessing the composition and structure of active species for Zr-catalyzed direct amide bond formations under operating conditions, we discovered zirconium oxo clusters form quickly and are likely active species in the reactions. Formation of these clusters dismisses the use of additional organic ligands, inert atmosphere, anhydrous solvents, or even water scavenging to provide amides in good to excellent yields. More specifically, dodeca- and hexazirconium oxo clusters (Zr_12_ and Zr_6_, respectively) rapidly form from commercial, readily available Zr salts under reaction conditions known to afford amides directly from nonactivated carboxylic acid and amine substrates. Extended X-ray absorption fine structure (EXAFS) experiments confirm the presence of oxo clusters in solution throughout the reaction, while their key role in the mechanism is supported by an in-depth computational study employing density functional theory (DFT) and molecular dynamics (MD) methods. These results underline the value of (earth-abundant) metals’ intrinsic solution chemistry to transformative mechanistic understanding and to enhance the sustainability of organic transformations.

## Abstract 2

Direct amide synthesis catalyzed by Zr salts is mediated by air and moisture-stable Zr^IV^ oxo clusters rapidly formed in situ, indicating a new strategy for taming the fickle reactivity and speciation of earth-abundant metals into well-behaved catalytic species.

## Introduction

Control over the solution chemistry of metal ions is vitally important for the design of efficient catalytic reactions. Generally, such control is achieved through organic ligands, which stabilize and govern the reactivity of a metal center. However, metal complexes commonly used as catalysts are often air and moisture sensitive, posing considerable challenges to both developing their reactivity and translating it into sustainable reaction conditions.^1,2^ This can be especially challenging for earth-abundant metals, whose reactivity is notoriously difficult to control. Herein, we introduce evidence supporting the involvement of zirconium oxo clusters formed in situ as catalytically active species in the direct formation of amide bonds catalyzed by Zr salts. These clusters form spontaneously from Zr salt precursors added at the beginning of the reaction, showcasing that fundamental knowledge of metal’s solution behavior is enabling in identifying alternative, more robust metal species that exhibit similar capabilities but provide unprecedented opportunities for developing reactions under more environment-friendly and cost-effective conditions.

Efforts to identify the active species of catalytic reactions frequently have a transformative impact on catalysis, enabling both much simpler conditions and the design of new reactions.^3,4^ Historically, catalysts based on group IV metals, alongside boron-based ones, have drawn considerable attention for the sustainable synthesis of amides directly from nonactivated carboxylic acid and amines.^5−12^ This route circumvents high energy demands, while preserving stereochemistry, and keeps waste to a minimum generating only water as a byproduct. However, these reactions remain sensitive to air and moisture due to the strong Lewis acid nature of the catalysts and thus require water removal techniques to preserve catalytic activity and to push reaction equilibrium toward products. These techniques often offset the benefits of catalysis, and unsurprisingly, metal-catalyzed amidations are still rare in industry.^13,14^ Recently, a rigorous investigation of a ZrCl_4_-catalyzed amide bond formation has shed light on several key details of the reaction mechanism, but the catalytically active species’ structure remained elusive and could not be elucidated from the experimental data. Instead, density functional theory (DFT) calculations were used to propose a bimetallic Zr complex as the most probable active species. However, the strong tendency of group IV metals to form oxo clusters, and their potential relevance in the reaction, has not been considered in this or even other previous studies.^15^ In fact, the potential key role of metal oxo clusters in metal-catalyzed reactions has been rarely evoked.^16−18^

**Figure 1:**
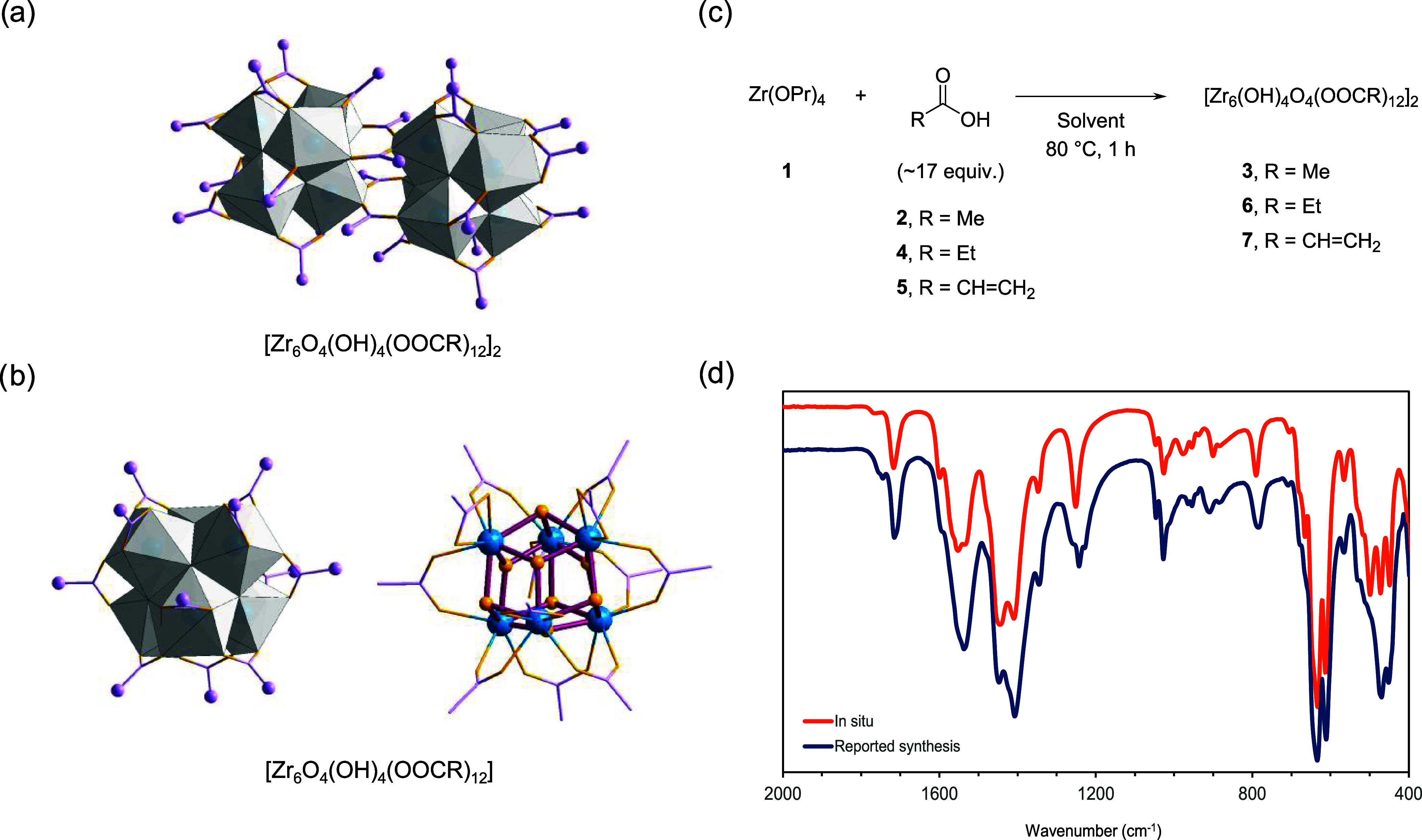
Zirconium oxo clusters (ZrOC) readily form when Zr(OPr)_4_ is mixed with carboxylic acids in organic solvents. Generic structures for (a) dodecanuclear and (b) hexanuclear ZrOC stabilized by carboxylate ligands, represented using a combination of polyhedron, wires/sticks, and ball-and-stick models. Gray polyhedron represents {Zr_6_O_8_}, light purple carbons, and gold yellow oxygens. (c) Reaction conditions used for obtaining ZrOC under *in situ* conditions previously developed for amide formation. (d) Comparison of infrared (IR) analysis of cluster [Zr_6_(OH)_4_O_4_(OOCCH_3_)_12_]_2_ (**3**) obtained *in situ* with a batch obtained following previous literature reports (reported synthesis).

In general, zirconium oxo clusters (ZrOC) are discrete species formed by the hydrolysis of Zr(IV) cations in the presence of appropriate ligands, such as carboxylates or phosphonates,^19,20^ and while their structures have been well described, their catalytic reactivity remains scarcely investigated.^21^ Leveraging this rich and yet underexplored chemistry, we have recently reported dodeca- (Zr_12_) and hexanuclear (Zr_6_) zirconium oxo clusters (ZrOC, Figure 1a,b) as new catalysts for the direct synthesis of amides.^22,23^ Zr_12_ and Zr_6_ oxo clusters exhibit many similarities in terms of structure and solution behavior. They share a common {Zr_6_O_8_} structural motif: while Zr_6_ clusters consist of one {Zr_6_O_8_} unit, Zr_12_ clusters exhibit two of these units linked by either an O/OH or, as in this study, carboxylate ligand bridges. These bridging ligands are less labile than those on the periphery of a Zr_12_ cluster, which in turn exchange easily with other coordinating species in solution.^24,25^ Remarkably, both clusters afforded a variety of amides without requiring anhydrous conditions or water scavenging techniques to afford products with good yields. Intriguingly, control experiments using Zr(OPr)_4_ generated products with similar efficiency to ZrOC, while other salts presented much lower reactivity, leading us to hypothesize ZrOCs are forming *in situ*, and could be acting as the real catalytic active species in Zr(OPr)_4_ solutions.

Herein, we not only show that ZrOCs are formed in high yields in relevant reaction conditions but also prove that their presence directly correlates with reaction efficiency. Further, analysis of reactions through extended X-ray absorption fine structure (EXAFS) and density functional theory (DFT) calculations corroborates their role as key catalytic species in the formation of amides under rather straightforward conditions. At large, these results advocate for exploiting the intrinsic solution chemistry of (earth-abundant) metal salts under experimental operating conditions to assess the composition and structure of active species and achieve unprecedented mechanistic understanding with the potential to enhance the overall sustainability of organic transformations. Our findings imply that discrete ZrOC might be the link between the solution chemistry of Zr(IV) and the mechanism of several organic reactions catalyzed by its metal salts, providing new ground for more efficient reactions to be carried out under simpler conditions. In the case of amide bond formations, this could mean performing reactions without industry-limiting water scavenging and in nonanhydrous reaction conditions.

## Results and Discussion

Intrigued by the similar yields of amide product obtained using either ZrOC or Zr(OPr)_4_ salt as a catalyst in our previous work,^22,23,26^ we decided to investigate the nature of the active catalytic species in these reactions. For that, we probed the formation of ZrOC using only acetic acid (**2**) and Zr(OPr)_4_ (Figure 1c). The reactions were conducted at 80 °C for 1 h using ethanol as the solvent. A ratio of AcOH/[Zr] = 100:6 and a reaction concentration of [**2**] = 3.3 M were used to allow for direct comparison with our amide formation reactions previously reported under similar conditions.^22,23^ After 1 h, a white solid was formed and was collected by centrifugation, dried under high vacuum, and analyzed by infrared spectroscopy (IR) (Figure 1d).

IR analysis and comparison with previously reported literature unequivocally confirmed the formation of [Zr_6_(OH)_4_O_4_(OOCCH_3_)_12_]_2_ (**3**) in >99% yield. In the IR spectrum, the characteristic bands of propoxide groups linked to zirconium atom at 1035–1135 cm^–1^ disappeared, indicating the propoxide ligands bound to the Zr atoms were fully replaced (Figure 1d). The vibrations at 1406, 1536, and 1718 cm^–1^ are consistent with the presence of carboxylate groups. The positions and frequency separation (Δυ = 130 cm^–1^) of the first two are typical of a bidentate carboxylate group coordination to metal centers in bridging position, while the latter can be assigned to carboxylates of noncoordinated molecules of **2**.^25,27^ In addition, the bands around 500 and 700 cm^–1^ were consistent with Zr–O_ligand_ and Zr–O–Zr bonds.^28^ Further comparison with the literature indicated the formation of [Zr_6_(OH)_4_O_4_(OOCCH_3_)_12_]_2_ (**3**).^24^ Reproducing the literature synthesis, and comparing the IR and NMR of solids obtained through both syntheses, unambiguously confirmed the formation of **3** in our reaction;^25^ the IR spectra of both batches overlap each other almost perfectly (Figures 1d and S1).

Cluster formation under amidation-relevant conditions appears to be a rather general phenomenon, as evidenced by reproducing initial experiments in different solvents and using different carboxylic acids. By applying the same initial strategy, the formation of ZrOCs using other carboxylic groups could also be observed under similar conditions. More specifically, propionic acid (**4**) and acrylic acid (**5**) also afforded the *in situ* quantitative formation of dodecanuclear ZrOCs [Zr_6_O_4_(OH)_4_(OOCCH_2_Me)_12_]_2_^24^ (**6**) and [Zr_6_O_4_(OH)_4_(OOCCH=CH_2_)_12_]_2_^29^ (**7**), respectively, as confirmed by comparison of IR and hydrogen nuclear magnetic resonance (^1^H NMR) of the solid obtained with samples prepared according to literature procedures (see SI for details). Furthermore, the facile formation of dodecanuclear ZrOC (Figure 1a) was also observed in different solvents. As Zr-catalyzed amidations are frequently conducted in solvents such as 1,4-dioxane, acetonitrile, and toluene, we tested whether any clusters would be formed in these solvents. Following the same approach used for the reactions in ethanol, **3** could be isolated in all cases (Figure S1). In 1,4-dioxane, the corresponding ZrOCs could also be obtained using acids **4** and **5** as confirmed by IR (Figures S3 and S5) and ^1^H NMR (Figures S2 and S4). The successful formation of ZrOC in other typical solvents used for amidations shows the reactivity is not limited to acetic acid but is likely a general feature of these systems. This generality is consistent with the several types of carboxylate ligands encountered in previously reported ZrOC structures.^19,20^

The nature of the Zr precursor influences ZrOC formed, as chloride-containing ZrCl_4_ and ZrOCl_2_ lead to the formation of distinct clusters from the ones formed from Zr alkoxides. In addition to Zr(OPr)_4_, other Zr salts commonly used in amidations were probed to check if they also provide a ZrOC under the same conditions.^30^ Unsurprisingly, Zr(OBu)_4_ behaved similarly to Zr(OPr)_4_ and afforded ZrOC **3** when it was reacted with acetic acid in EtOH at 80 °C for 1 h (Figure S7). This suggests Zr alkoxides generally react the same way, likely due to the quick formation of Zr(OEt)_4_ in both cases.^31^ On the other hand, IR of the solids obtained using ZrCl_4_ and ZrOCl_2_ under the same reaction conditions clearly showed that both precursors afforded the same product, but a distinct one from the clusters obtained using Zr alkoxides (Figure S8). The characteristic bands of carboxylates coordinated to Zr (around 1500 cm^–1^) and Zr–O/Zr–O–Zr bonds (region between 750 and 500 cm^–1^) present in their IR spectra strongly suggested this new product was also a ZrOC.

Since many Zr-based metal–organic frameworks (Zr-MOFs) featuring [Zr_6_O_8_] are synthesized using either ZrCl_4_ or ZrOCl_2_, we hypothesized the solids obtained from these Zr precursors could be hexanuclear clusters with a general structure as the one presented in Figure 1b.^21^ Evidence in support of this hypothesis was obtained through the comparison of IR spectra of unknown solids obtained from chlorinated Zr salts with those from previously reported [Zr_6_O_4_(OH)_5_(C_2_H_3_O_2_)_8_(H_2_O)_2_Cl_3_] (**8**) (Figure S8).^32^ The IR spectra for all three batches of compounds were exactly the same in the 4000–400 cm^–1^ region, while the far IR (FIR) between 400 and 300 cm^–1^ indicated the presence of Zr–Cl bonds,^33^ thus suggesting **8** was formed when ZrCl_4_ and ZrOCl_2_ were used as Zr salts in the conditions reported in this study (Figure S8). The reasons behind the influence of chloride in the nuclearity of the cluster obtained in this case are yet elusive, and more experiments are certainly needed to elucidate how the precursor’s nature affects cluster formation. However, these results are consistent with the formation of Hf_6_ oxo clusters from HfCl_4_ at low temperatures reported recently, while dodecanuclear clusters were only observed when temperatures surpassed 120 °C.^34^

Considering that the formation of a ZrOC cluster under conditions relevant for amidation reactions could be clearly observed, we further investigated their relevance for the reaction performance (Table 1). Thus, a series of experiments was performed comparing the reaction yields when amine was added at the beginning of the reaction (method A), or after 1 h of stirring a mixture consisting of acetic acid and Zr salts (method B). In this way, we could probe indirectly if allowing ZrOC to form before amine addition has any benefit for the reaction, thereby showing the relevance of ZrOC for the yield of amide product obtained. Acetic acid (**2**) and benzylamine (**9**) were employed as model substrates to probe the reaction outcome using either Zr(OPr)_4_ or ZrCl_4_ as catalyst precursors. The reactions were carried out following our previously optimized conditions.^22,23^

**Table 1:**
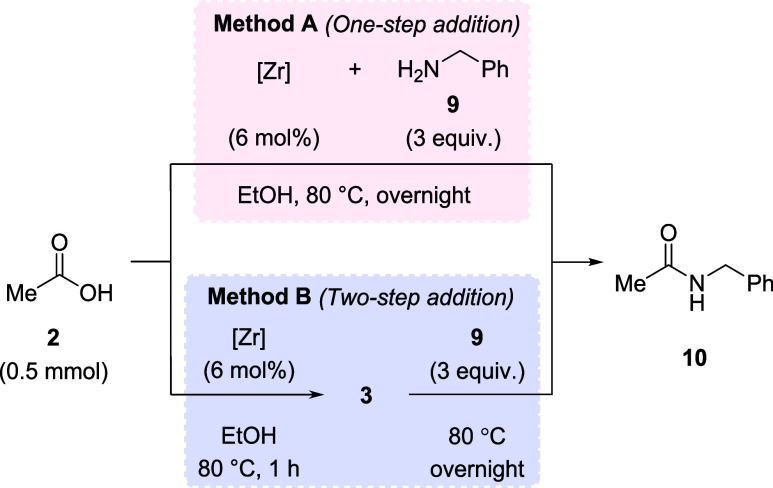
Catalytic Relevance of ZrOC Formed In Situ

entry	method	[Zr]	solvent	yield (%)^a^
1	A		EtOH	2
2	A	Zr(OPr)_4_	EtOH	59
3	B	Zr(OPr)_4_	EtOH	73
4^b^	B	Supernatant from the synthesis of **3**	EtOH	21
5	A	ZrCl_4_	EtOH	61
6	B	ZrCl_4_	EtOH	74
7	A	Zr(OPr)_4_	1,4-dioxane	91
8	A	Zr(OPr)_4_	DMSO	100
9^c^	B	Zr(OPr)_4_	EtOH	87

a ^1^H NMR yields.

b Reaction centrifuged after the first step.

c Gram-scale reaction using 10 mmol of **2**.

In ethanol, similar yields were observed using methods A and B for both Zr(OPr)_4_ and ZrCl_4_ (Table 1, entries 2 vs 3 and 5 vs 6), although the yields for method B are slightly higher than for method A. In addition, two control experiments were performed. In the absence of Zr(OPr)_4_ species, negligible product yield was obtained, confirming the Lewis acidity of Zr(IV) promotes the reaction (Table 1, entry 1).^22,23^ Additionally, using Zr(OPr)_4_, we repeated the reaction employing method A. In this control reaction, the cluster that formed after the first hour was separated from the reaction by centrifugation (Table 1, entry 4). Inductively coupled plasma analysis of the supernatant revealed that only 2.3% of the initially added zirconium remained in solution after cluster formation (9.3 μmol). In this case, amide **10** was formed in only 21% yield, confirming that when the initially formed cluster is removed, a sharp drop in reaction yield occurs. Good yields were also observed in nonalcoholic solvents such as 1,4-dioxane and dimethyl sulfoxide (DMSO), which we have shown previously to afford amides in good yields using preformed ZrOC (Table 1, entries 7 and 8). Finally, a gram-scale reaction also afforded excellent yield (Table 1, entry 9), showcasing that the method is not restricted to small-scale reactions. Together, these results strongly suggest that clusters are formed in the reaction and that they play a key role in forming the amide product.

### Mechanistic Investigations

As the evidence presented above showed that cluster formation is a rather general phenomenon for several conditions in which Zr(IV)-catalyzed amidations are carried out, we attempted to further interrogate the reaction mechanism using extended X-ray absorption fine structure (EXAFS) and density functional theory (DFT) calculations. Taking into account the reactivity pattern observed for experiments in Table 1, and our previously published solution NMR studies and reactivity results,^22,23^ we hypothesized [Zr_6_O_8_] clusters or their dimeric [Zr_6_O_8_]_2_ structures could be acting as active species. To corroborate that and elucidate key molecular features of this unexpected class of catalysts, we have analyzed reaction mixtures using EXAFS to confirm the presence of clusters in solution and how the inorganic [Zr_6_O_8_] core unit is affected throughout the reaction. These investigations used preformed dodeca-^22^ and hexanuclear^23^ Zr oxo clusters and phenylacetic acid (**11**) and benzylamine (**9**) as substrates to link the previously disclosed results with the findings in this study. Furthermore, we performed DFT calculations and molecular dynamics (MD) simulations to gain mechanistic insights into the nature of Zr oxo cluster catalysts for direct amidation and the behavior of the catalysts in solution.

#### EXAFS Analysis

**Figure 2:**
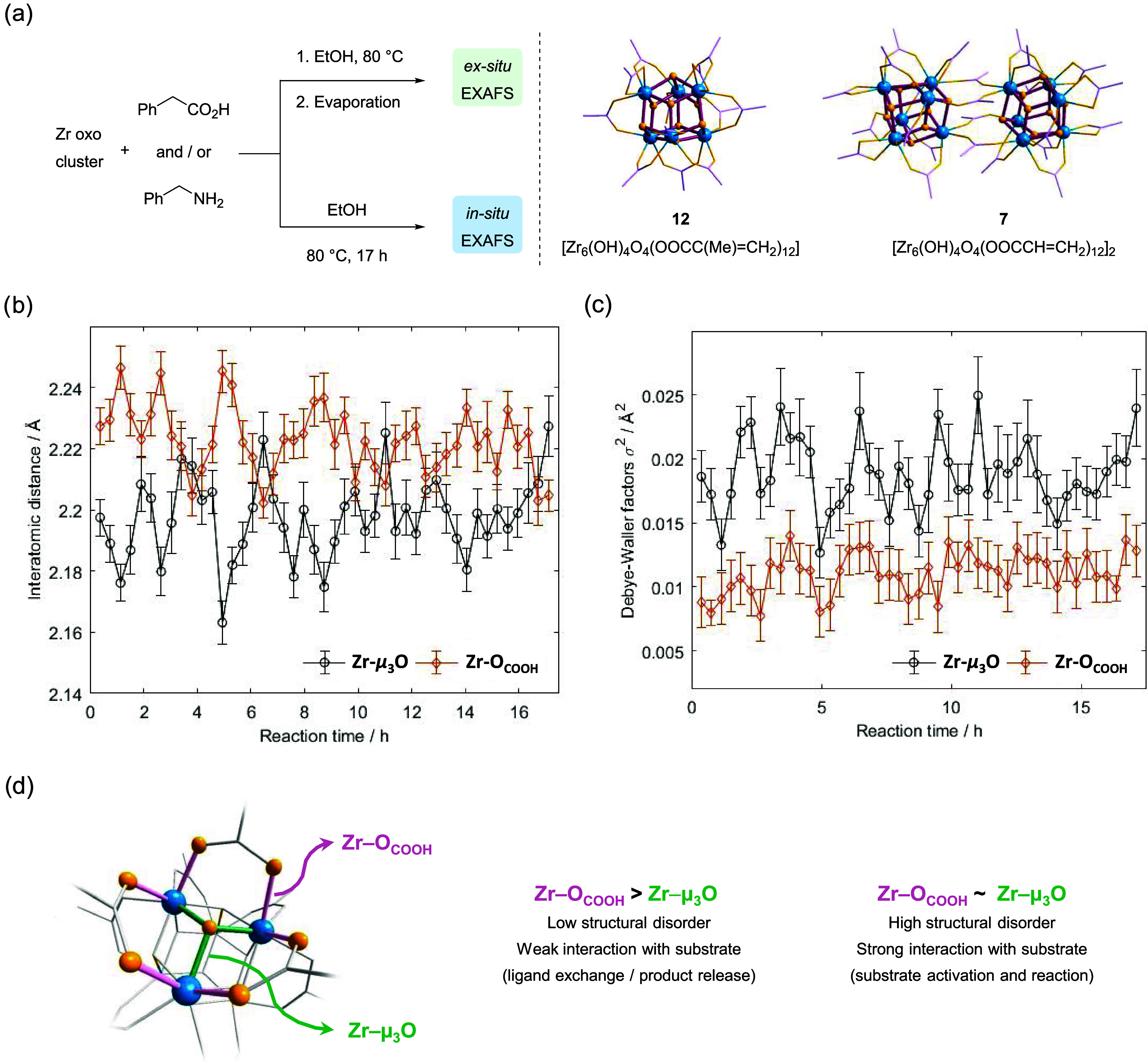
EXAFS analysis shows the cluster remains stable throughout the reaction, but it contracts and expands due to changes in its coordination sphere. (a) Workflow for the preparation of samples for EXAFS using Zr_6_ cluster **12** or Zr_12_ cluster **7** (see SI for details). Illustrative structures for both clusters are given using a combination of wires/sticks for carboxylate ligands (structure simplified for clarity) and ball-and-stick models for the {ZrO_8_} core; blue represents zirconium, light purple carbons, and gold yellow oxygens. (b) Variations of Zr–O_COOH_ and Zr-μ_3_O bond lengths and (c) respective trend in Debye–Waller factors as a result of structural disorder observed by in situ EXAFS analysis of the amidation reaction between phenylacetic acid (**11**) and benzylamine (**9**) catalyzed by Zr_12_ cluster **7** (error bars for confidence interval of 95%). Spectra 1 and 2 consist of a mixture of **7** and **11**, while the addition of **9** occurred in correspondence to spectrum 3. (d) Summary of main results for the variations in Zr–O_COOH_ and Zr-μ_3_O bonds observed by in situ EXAFS analysis of amidation reaction.

Initial EXAFS experiments at the Zr K-edge were performed using *ex situ* solid samples prepared by mixing the Zr_12_ cluster **7** (acrylate capping ligands) and the Zr_6_ cluster [Zr_6_O_4_(OH)_4_(OOCC(CH_3_)=CH_2_)_12_]^35,36^ (**12**) featuring methacrylate capping ligands with acid **11** and amine **9** (Figure 2a). Formation of **12** in relevant amidation conditions proceeds analogously to that observed for other carboxylic acids (see Experimental Section for details). To understand how the cluster might be affected by both reagents, we looked into the average variation of Zr-μ_3_O(H) bonds, which are part of the inorganic [Zr_6_O_8_] core, and Zr–O_COOH_, which refers to Zr-capping ligand interactions (Figure 2d). Importantly, when amine is present, the Zr–O_COOH_ fragment comprises both Zr–O and Zr–N bonds, given the negligible difference in the backscattering amplitude of consecutive light elements. Furthermore, two types of Zr-μ_3_O bonds are present in the [Zr_6_O_8_] core, namely, Zr-μ_3_OH and two Zr-μ_3_O; however, as EXAFS affords only average atomic distances on the environment surrounding the metal centers being analyzed, to consider two different signals arising from these moieties would be an overinterpretation of the data. Therefore, to analyze the EXAFS data obtained, we have relied on a simple yet robust model of the Zr_6_O_8_ catalytic cluster (Figure S9b), which allowed us to confidently retrieve each structural parameter reported, including interatomic distances, with careful and accurate statistical analysis (see Experimental Section for details). This allowed us to interpret structural variations to the cluster during the reactions safely on the basis of the data obtained.

Both clusters exhibit interesting distinct behavior in the presence of acid **11** and amine **9** (Figure S10). For the Zr_6_ cluster **12**, the Zr-μ_3_O bonds remain virtually unaltered in all samples, while Zr–O_COOH_ bonds lengthen in the presence of acid **11**, in line with a favorable ligand exchange detected by nuclear magnetic resonance (NMR) in our previous study.^23^ On the other hand, both Zr–O_COOH_ and Zr-μ_3_O bonds of Zr_12_ cluster **7** increase when **11** is added, suggesting a greater dynamic for this cluster compared with cluster **12**. For both clusters, the concomitant addition of amine **9** and acid **11** does not cause major structure changes with respect to the cluster on its own, though the addition of **9** to a mixture of cluster and **11** affects more the Zr–O_COOH_ bonds in Zr_6_ than in Zr_12_. These structural fluctuations are also evident in the disorder associated with interatomic distances (Figure S10). The apparent greater dynamic nature of the Zr_12_ cluster compared with Zr_6_ is in line with our previous hypothesis attributing the slightly higher reactivity of Zr_12_ to the greater lability of its coordination sphere.^23^

In addition to *ex situ* samples, we have also followed the changes in the EXAFS Zr K-edge signal *in situ* using the amide formation between **9** and **11** catalyzed by Zr_12_ cluster **7** in EtOH at 76 °C as a model reaction (Table S2). Both the generality of the reaction with respect to various reaction conditions and the better reactivity observed with the Zr_12_ cluster compared with Zr_6_ ones reported previously^23^ supported the representative character of this model reaction. This choice also largely streamlined our experimental design. The reaction was followed by 17 h, collecting one spectra every ∼23 min, and variations on the average length of Zr–O_COOH_ and Zr-μ_3_O bonds, as well as Zr–Zr second-shell distances, could be observed over the course of the reaction (Figure S11). In general, the excellent fit of the EXAFS model to the experimental results shows no other types of clusters are present during the reaction, such as a trinuclear cluster^37^ or a distinctive dodecanuclear cluster where the Zr_6_O_8_ units are bridged by the O/OH group.^38^ Furthermore, cluster **7** clearly retains its structure throughout the reaction, as evidenced by the constant Zr–Zr second-shell distances, signal degeneration, and corresponding small variation of Debye–Weller factors observed (Figures S11 and S12). Together with the low reaction yield obtained when removing clusters formed *in situ* (entry 4, Table 1), these EXAFS results support the preponderant role of Zr_6_O_8_ cluster units in the reactions, as opposed to other cluster species that could potentially be formed.^19,34^

*In situ* EXAFS analysis has also shown that the reaction affects the Zr first-shell environment, revealing that the changes in Zr–O_COOH_ and Zr-μ_3_O bonds are concomitant and in opposite directions (Figure 2b,c). This pattern suggests that the inorganic core of the cluster goes through a series of contractions and expansions during the reaction to compensate for the changes occurring in the external coordination sphere caused by the catalytic process (Figure 2d). This dynamic is also coherent with variations in Debye–Waller (DW) factors observed for Zr–O_COOH_ and Zr-μ_3_O bonds along the reaction. In this case, we considered the DW factors to be directly correlated to structural disorders induced by the reaction, as measurements were performed at constant temperature.^39^ During the reaction, the DW factors related to Zr–O_COOH_ and Zr-μ_3_O bonds varied concomitantly and synchronously (Figure 2c). The higher structural disorder of the Zr-μ_3_O fragment was attributed to the dual Zr-μ_3_O(H) nature of this group. Based on these variations, the magnitude of structural disorders seems to increase when Zr–O_COOH_ ∼ Zr-μ_3_O (compare Figure 2b,c). This could arise from a strong interaction of substrate with a Zr site, presumably related to the activation of substrate, as expected in a Lewis acid-catalyzed reaction. Accordingly, the magnitude of induced structural disorder seems to decrease when Zr–O_COOH_ ≫ Zr-μ_3_O, which would be consistent with weak ligand–cluster interactions, characteristic of ligand exchange or product release steps (Figure 2d).

### Computational Study

Prompted by the experimental evidence strongly supporting the involvement of ZrOCs in direct amide formation reactions, we also studied this reaction through DFT calculations. In a previous contribution, Adolfsson, Himo, and co-workers showed that dinuclear Zr species enable lower energy barriers for amidation reactions than mononuclear species, pointing to dinuclear species as actual catalytic species in direct amidations catalyzed by Zr salts.^15^ Thus, we propose here that these clusters are in fact formed under reaction conditions and that they catalyze the amide formation more effectively than the mononuclear Zr precursors due to a cooperative action of binuclear Zr^IV^–Zr^IV^ sites.

To confirm this hypothesis, an in-depth mechanistic investigation using DFT calculations was carried out for the amide bond formation between phenylacetic acid (**11**) and benzylamine (**9**) catalyzed by representative Zr species. More specifically, [Zr_6_O_4_(OH)_4_(OOCC(CH_3_)=CH_2_)_12_] cluster (**12**) was chosen as a model ZrOC given its proven versatility in catalyzing amide formation for a variety of substrates.^23^ Cluster **12** catalyzed the reactions in essentially the same conditions as Zr_12_ cluster **7**, thus allowing us to carry out our mechanistic study at a much lower computational cost. For a mononuclear Zr species, we opted for complex [ZrCl_3_(OOCC(CH_3_)=CH_2_)(BnNH_2_)] (**M**) as a model, based on previous computational findings.^15^ Importantly, the stability of Zr_6_ oxo cluster **12** under current experimental conditions has been theoretically corroborated by estimating the energetics of cluster formation and decomposition (see the Supporting Information for a detailed discussion). In short, the formation of Zr_6_ cluster **12** from a mixture of Zr(OPr)_4_ and methacrylic acid in DMSO through esterification reactions^37^ is highly exergonic (−201.3 kcal mol^–1^) and more thermodynamically favorable than that of analogous Zr_3_, Zr_4_, and Zr_5_ oxo clusters (Table S3). Similarly, the decomposition of cluster **12** via hydrolysis and alcoholysis would require a large energy input (>38 kcal mol^–1^, Table S4). These results corroborated the stability previously inferred experimentally^23^ and confirmed that a hexanuclear Zr species is the main species present in solution when cluster **12** is used as a catalyst.

**Figure 3:**
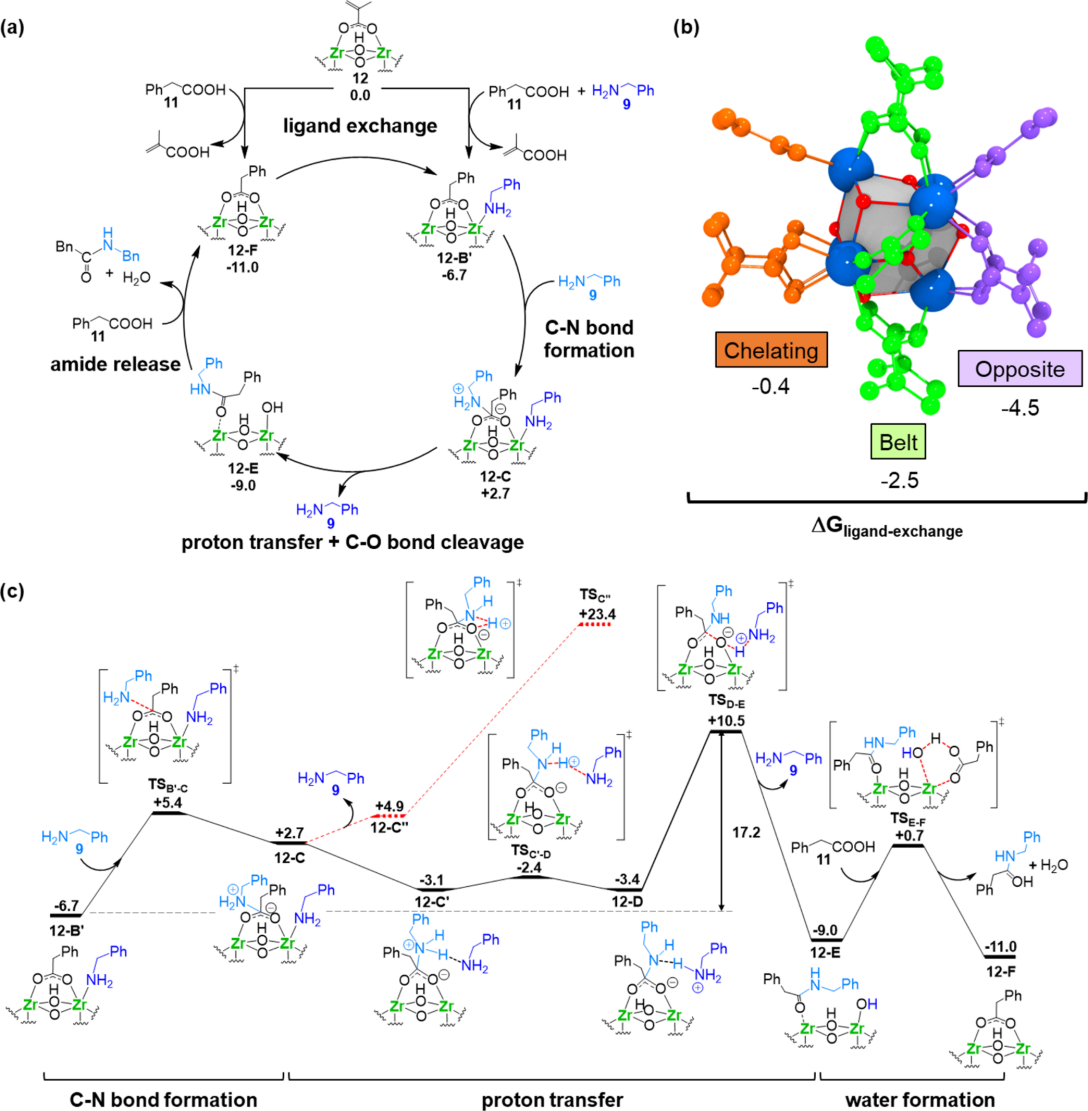
DFT calculations corroborate the feasibility of ZrOC as active species in the direct amide formation catalyzed by Zr salts. (a) Proposed catalytic cycle for the amide bond formation between phenylacetic acid **11** and benzylamine **9** catalyzed by Zr_6_ cluster **12**; DFT computed relative Gibbs free energies in kcal mol^–1^. (b) 3D representation of Zr_6_ cluster **12**, highlighting the different coordination sites: “opposite” (purple), “belt” (green), and “chelating” (orange). (c) Gibbs free-energy profile for the mechanism of the amide bond formation between phenylacetic acid **11** and benzylamine **9** catalyzed by **12**; relative free energies referred to **12** and the free substrates in kcal·mol^–1^.

For our study, we initially proposed a four-step catalytic cycle (Figure 3a). This reaction pathway was devised based on mechanistic experiments (e.g., NMR studies, control experiments, etc.) we carried out using preformed clusters in our previous study^22,23^ and on previous mechanistic studies of amide bond formation or hydrolysis by Zr-MOFs,^26,40^ Zr-salts,^15^ and Zr-containing metal oxo clusters.^41−43^ The reaction likely starts with (i) an exchange of a capping methacrylate ligand by an acid substrate molecule, followed by (ii) a nucleophilic attack of an amine to the acid substrate activated by a Zr(IV) center, leading to the formation of a C–N bond and resulting in a well-defined intermediate with the attacked carbon exhibiting a tetrahedral sp^3^ hybridization.^15^ Next, (iii) N,O-proton transfers would trigger the C–O(H) bond cleavage, forming the amide product and a Zr–OH group, and then (iv) complexation of a new molecule of the acid substrate releases the reaction’s product and regenerates the catalyst, thus closing the cycle (Figure 3a). Importantly, the initial ligand-exchange step is bypassed when the ZrOC catalyst is formed in situ, as the capping carboxylate ligands are substrates themselves. However, this step is still essential to enable the catalytic activity of ZrOC toward acids distinct from capping ligands. Thus, to build a complete mechanistic picture that would also inform our previous works,^22,23^ we included it in our study.

#### Cluster Model System

As the starting model for cluster **12**, we employed the corresponding crystal structure,^23^ which features three distinct coordination environments, labeled as “opposite”, “belt”, and “chelating”, differing in the coordination mode of carboxylate ligands and local environment (Figure 3b). Thermodynamically, the exchange of methacrylate by phenylacetic acid is more favorable at the “opposite” site than for other sites (Δ*G*_ligand-exchange_ = −4.5, −2.5, and −0.4 kcal·mol^–1^ for “opposite,” “belt,” and “chelating,” respectively). Thus, we continued our study assuming that the reaction starts by ligand exchange at the “opposite” site of cluster **12**. In addition, we employed a catalyst model that simplifies some of the methacrylate ligands by formate at remote positions of the active site for the sake of computational efficiency (Figure S14, see the Supporting Information for full discussion).

#### Ligand Exchange

In essence, the exchange mechanism of methacrylate by phenylacetic acid follows a similar principle to those previously proposed based on computational and variable temperature NMR studies, where the incoming acid is deprotonated while the outgoing carboxylate is protonated.^44,45^ In this process, the original carboxylate ligand has been proposed to first shift from a bridging to a monodentate (or chelating) coordination mode, opening a nearby vacant site able to receive the incoming carboxylate moiety. Our calculations in the absence of amine are in line with this previous mechanistic picture (Figure S16). However, unlike previously reported, we found that amine coordination promotes ligand exchange by significantly decreasing the overall free-energy barrier in 5.2 kcal mol^–1^ (compare amine-assisted and direct ligand-exchange mechanism in Figures S15 and S16, respectively). The coordination of a benzylamine **9** to one of the Zr centers of **12** (**12-A**) triggers a change in the methacrylate ligand coordination mode from bidentate to monodentate affording species **12-A′**, which features a H-bonding interaction between the decoordinated carboxylate oxygen and a proton of the amine. From monodentate species **12-A′**, phenylacetic acid **11** can replace the monodentate methacrylate through an affordable free-energy barrier of 16.9 kcal mol^–1^, whereby the ligand exchange occurs concomitantly with a proton transfer from the incoming phenylacetic acid (**11**) to the leaving methacrylate acid in a six-membered transition-state structure (Figure S17). The resulting intermediate **12-B** lies 6.7 kcal mol^–1^ below the reactants in terms of free energy and was found to be in equilibrium with the isoenergetic **12-B′**, in which the carboxylate group of the substrate binds two Zr centers of ZrOC in a bridging fashion. These findings are supported by previous NMR studies, which already inferred that benzylamine could spontaneously bind to Zr centers of ZrOCs,^23^ as well as by crystallographic structures of hexanuclear ZrOCs where coordination of propanol to a Zr site is also accompanied by monodentate coordination of a carboxylate ligand at the neighboring Zr center.^29^ They are also in line with the beneficial effect of an excess of amine observed in our previous study.^22,23^

#### Amide Product Formation

Figure 3c shows the free-energy profile for the mechanism of amide bond formation starting in intermediate **12-B′**, in which a phenylacetate molecule binds in bidentate fashion bridging two Zr atoms of the cluster. In this interaction, the carboxylate group is activated by the Lewis acidity of the two Zr(IV) sites, enabling the nucleophilic attack of an external amine on the carboxylic carbon to yield C_acid_–N_amine_ bond formation and C–O bond cleavage. Thus, species **12-B′** is more reactive than its isomer **12-B**, in which the carboxylate binds only to one Zr(IV) site (see above). This relevance of the Zr(IV) Lewis acidity on the reaction is also in line with our experiments in Table 1 and prior literature, where very low amide yields are obtained in the absence of a strong Lewis acid activation.^15,30,46^ The outer-sphere nucleophilic attack, which is analogous to that observed for Zr(IV)-catalyzed peptide hydrolysis,^40,41,47^ occurs through a low free-energy barrier of 12.1 kcal mol^–1^ (**TS**_**B′-C**_ in Figure S17). The resulting metastable zwitterionic intermediate **12-C** rapidly converts to the more stable species **12-C’**, in which the N atom of the formerly coordinated amine interacts with an N-bound proton of the zwitterionic moiety forming a hydrogen bond (see dark blue amine in species **12-C′** of Figure 3c).

Next, from **12-C′**, we propose a stepwise, proton-transfer process assisted by the free amine nearby acting as a proton shuttle and the C–O(H) bond breaking to yield the coordinated amide product (**12-C′** → **12-D** and **12-D** → **12-E** in Figure 3c). The second proton transfer inducing C–O(H) bond cleavage occurs through transition-state **TS**_**D-E**_, which is the rate-determining transition state of the process, and results in an overall free-energy barrier of 17.2 kcal mol^–1^ (**12-B′** → **TS**_**D-E**_), a moderate value which can be easily overcome under the reported experimental conditions. The formation of Zr-hydroxo/Zr-amide intermediate **12-E** is exergonic (9.0 kcal mol^–1^ below reactants) and provides the thermodynamic driving force to the reaction. In the final step, the amide product is released to the solution, and the active form of the catalyst with a phenylacetate ligand coordinated (**12-F**) is regenerated. Thus, a new molecule of acid substrate **11** transfers a proton to the Zr–OH moiety forming the corresponding phenylacetate, which displaces the coordinated amide and releases a water molecule. This step proceeds through the transition-state **TS**_**E-F**_ and a low free-energy barrier (Δ*G*^‡^ = 9.7 kcal mol^–1^). Finally, the coordination of benzylamine to **12-F** yields species **12-B′**, closing the catalytic cycle.

#### Additional Considerations about the Catalytic Cycle

We also explored other mechanisms for amide formation by Zr_6_ cluster **12**. First, from intermediate **12-C**, the proton transfer to the carboxylate oxygen could occur directly, without the assistance of an addition amine molecule (red dashed lines in Figure 3c). This pathway is unlikely, as it would require a direct proton-transfer step through a strained four-membered ring transition-state geometry (**TS_C″_**) with a high, overall free-energy barrier of 30.1 kcal mol^–1^. Second, the formation of a C–N bond could occur through an inner-sphere nucleophilic attack of the Zr-amido intermediate resulting from amine coordination and deprotonation by the carboxylate (Figure S18). Nevertheless, the Zr-amido species is computed by a high-energy laying intermediate (23.4 kcal mol^–1^ above reactants), even higher than the rate-determining transition state for the amine-assisted mechanism (**TS**_**D-E**_). Moreover, in our mechanistic proposal, the amine-assisted proton-transfer steps to induce the C–O(H) bond cleavage was identified as the rate-determining step of the whole reaction, which is consistent with the experimentally observed acceleration of the reaction rate as the concentration of amine increases.^22,23^

To link the complete mechanistic picture discussed above with the reactions using Zr salts and nonactivated carboxylic acids, where Zr clusters are formed in situ, we probed the feasibility of amidation of benzylamine (**9**) with acetic (**2**), acrylic (**5**), and methacrylic acids catalyzed by ZrOC capped with the corresponding carboxylate ligands. To this end, we computed the key steps of the mechanism using [Zr_6_O_4_(OH)_4_(OOCMe)_12_] (**3m**), [Zr_6_O_4_(OH)_4_(OOCCH=CH_2_)_12_] (**7m**), and [Zr_6_O_4_(OH)_4_(OOCC(CH_3_)=CH_2_)_12_] (**12**) hexanuclear clusters as model catalysts (Figures S18 and S19). In all cases, the computed overall free-energy barriers from amine adduct intermediate **B′** to the transition state involving the final C–O bond cleavage (**TS**_**D-E**_) show moderate values ranging from ca. 14 to 17 kcal mol^–1^. These results underline the feasibility of direct amide formation on substrate-decorated ZrOCs formed in situ from Zr salts and further validate our proposed mechanistic picture in Figure 3c. Interestingly, the computed reactivity trend acetic acid > methacrylic acid > acrylic acid (Δ*G*_overall_^‡^ = 13.9, 15.5, and 16.6 kcal mol^–1^, respectively) follows the same trend as the basicity of the corresponding carboxylates (p*K*_a_ for the corresponding conjugate acids: 4.76, 4.65, and 4.25, respectively),^48^ in line with the proposed rate-determining transition state (**TS**_**D-E**_) where the oxygen atom of the carboxylate captures a proton concomitantly to the C–O(H) bond cleavage. In this case, the reactivity is not governed by the electrophilicity of the carboxylate carbon since the nucleophilic attack of the amine is not involved in the rate-determining process.

Finally, we have also computed the catalytic cycle for amide bond formation between the benzylamine (**9**) and the methacrylic ligand in the [ZrCl_3_(OOCC(CH_3_)=CH_2_)(BnNH_2_)] mononuclear species (Figure S21). The reaction steps are similar to those of catalyst **12** (Figure 3a), except for the proton transfer assisted by the free amine to the carboxylic oxygen and the C–O(H) bond cleavage, which occurs in a stepwise manner. The latter process, involving C···O bond breaking and formation of the Zr-hydroxo moiety, is the rate-limiting step for mononuclear catalyst **M**. More importantly, the computed overall free-energy barrier with model mononuclear complex **M** (23.3 kcal mol^–1^) is significantly larger than that computed for hexanuclear catalyst **7m** (15.5 kcal mol^–1^). This indicates that the formation of Zr oxo clusters is beneficial for the reaction, presumably due to two adjacent Zr(IV) atoms cooperating in the coordination and activation of the carboxylic acids.

#### Insights on the Cluster Solution Behavior from Molecular Dynamics

**Figure 4:**
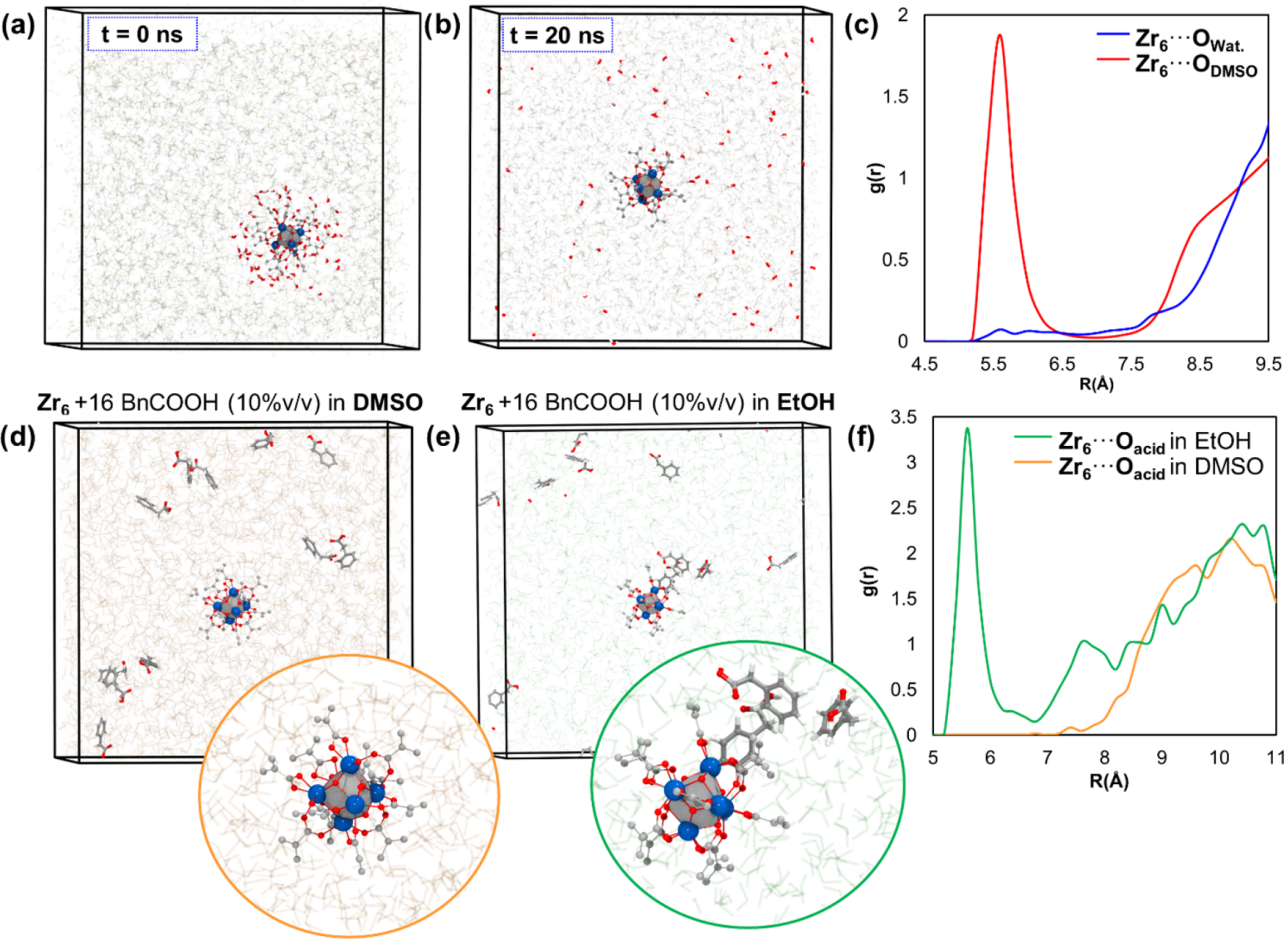
Molecular dynamics simulations of the ZrOC **12** catalyst in solution. Top panel from left to right: (a) Initial configuration of simulation box for **12** in DMSO with 10% v/v of H_2_O placed around the Zr cluster; (b) configuration after 20 ns of simulation at 343 K; and (c) radial distribution function (RDF) between the center of mass of the Zr cluster and the water oxygen (blue line) and DMSO oxygen (red line) averaged over the last 10 ns of the simulation. Botton panel from left to right: (d) Final snapshot of the simulation of **12** in DMSO with 10% v/v phenylacetic acid **11**, (e) final snapshot of the simulation of **12** in ethanol with 10% v/v phenylacetic acid **11**, and (f) RDF between the center of mass of the Zr cluster and the acetic oxygens (green line) and DMSO oxygen (orange line) averaged over the last 10 ns of the simulation.

Intrigued by the dismissal of water scavenging in amide bond formations catalyzed by Zr oxo clusters observed here and in our previous studies,^22,23^ we carried out atomistic molecular dynamics (MD) simulations to assess the affinity of ZrOC **12** catalyst toward water (a reaction product) and the reactants in DMSO and ethanol to better understand how the behavior of **12** in solution affects the reactivity. To this end, we first simulated **12** cluster in DMSO at different water concentrations and in both the presence and the absence of other species present in the reaction mixture (reactants and products). These simulations revealed the unfavorable nature of **12**···water interactions (Figures 4a–c, and S22–S25), as water molecules were more likely to follow Brownian motion in the solvent bulk rather than visiting the surface of **12**. In fact, test-case simulations starting from configurations where **12** is surrounded by water molecules, interacting through H-bonds with the basic oxygens of the ZrOC **12** cluster, show how these water molecules are spontaneously expelled from the solvation shell of the cluster, being replaced by DMSO molecules (Figure 4a,4b). Accordantly, the radial distribution function (RDF) of water molecules around ZrOC does not show any unambiguous peak, indicating the absence of a preferred contact distance (Figure 4c, blue line). On the other hand, RDF of oxygen atoms of DMSO around ZrOC shows a relatively sharp peak centered at ∼5.6 Å that integrates to approximately 3 DMSO molecules (Figure 4c, red line). This corresponds to DMSO molecules interacting with the Zr cluster through hydrogen bonds between the DMSO oxygen and the μ_3_–OH groups of the cluster as illustrated in the snapshot of Figure S23. Thus, these results suggest that the organic ligands of the cluster grant a hydrophobic environment to the catalytic sites, which might prevent the reverse amide hydrolysis and explain, in turn, why water scavengers are not required to observe amide formation in good yields when using ZrOCs as catalysts. This is also in line with our previous report reversing the hydrolytic reactivity^49^ of a ZrOC-based MOF toward amide bond formation activity by simply exchanging the reaction solvent.^26^

We have also compared the simulations of the ZrOC **12** catalyst with 10% v/v phenylacetic acid reactant (**11**) in DMSO and ethanol solvents. In DMSO, the acid molecules do not have a preferred interaction with cluster (Figure 4d), as observed also in RDF of acid molecules around **12** (Figure 4f, orange line). Conversely, the ethanol solvent does not disrupt the initial configuration of phenylacetic acid molecules surrounding the cluster, as evidenced by the presence of a sharp peak in RDF between the acidic oxygen and **12** (Figure 4e,f, red line). Thus, we hypothesize that ethanol cannot completely replace the initial carboxylic acid layer around ZrOC, hampering the access of amine reactants to the cluster inorganic core, which is a key process on amide formation, as shown by the DFT characterization of the reaction mechanism. This macroscopic behavior contributes to explain the lower yields in alcoholic solvents compared with DMSO observed experimentally (see above). In fact, previous experimental findings have revealed that additional carboxylic acid molecules are virtually impossible to eliminate during cluster synthesis.^25^

## Conclusions

In summary, our results identified the discrete zirconium oxo clusters (ZrOC) as key active species in direct amidation reactions catalyzed by simple, commercially available zirconium salts. The evidence reported here supports this conclusion in three convergent fronts: (1) ZrOCs are formed from simple Zr(IV) salts in high yield under relevant amidation conditions, providing amide products in good to excellent yields; (2) ZrOCs are stable throughout the reaction, as evidenced by the constant Zr–Zr interatomic distances and stable signal degeneracy for EXAFS signals measured during the reaction; and (3) corroboration of the energetic feasibility of catalysis by a computational study, which has also critically rationalized the benefit of an excess of amine to streamline proton-shuttling in the rate-determining C–O bond cleavage transition state. The formation of Zr oxo clusters *in situ* is beneficial for the reactivity since two Zr atoms can cooperate to coordinate and activate the carboxylic acids, reducing the energy barriers compared with mononuclear species. Also, the carboxylate ligands decorating the inorganic core create a hydrophobic cover that expels the water molecules produced during the reaction preventing the reverse peptide hydrolysis as revealed by dynamic simulation of bulk systems in solution. Remarkably, the nature of the rate-determining step allowed rationalizing reactivity trends as a function of the basicity of the acid substrate’s conjugated base. As acid substrates work also as capping ligands, this correlation outlines an exciting possibility of rationally fine-tuning catalytic activity by consolidated ligand design approaches. In concert with the versatility and synthetic utility demonstrated previously for zirconium oxo clusters,^22,23^ these results reveal that minute control of metal speciation state directly impacts reaction efficiency by enabling the formation of active well-defined and tunable catalysts suitable for reactions under air and moisture ambient conditions. As metal oxide structures are prevalent entities in nature, pursuing analogous connections for other metals could streamline the development of catalysts based on abundant, cheap, readily available, and sustainable metals, ultimately impacting many crucial synthetically useful organic reactions.

## Experimental Section

### General Remarks

Unless otherwise noted, reactions were performed without any precautions against air and moisture in 4 mL (1-dram) vials sealed with a PTFE-lined screw cap, and reagents were purchased from commercial sources and used as received.

Hydrogen nuclear magnetic resonance (^1^H NMR) was recorded on a Bruker Avance 300 (300 MHz) or a Bruker Avance 400 (400 MHz) spectrometer. Chemical shifts (δ) are reported in parts per million (ppm) downfield from tetramethylsilane and are referenced to resonance of residual solvent peak in the NMR solvent (^1^H NMR: DMSO-*d*_6_: δ = 2.50 ppm; CDCl_3_: δ = 7.26 ppm; and Toluene-*d*_8_: δ (CD_3_)) = 2.08 ppm. Fourier transform infrared spectra (FTIR) were recorded on a Bruker Vertex 70 spectrometer and analyzed with the Bruker OPUS software (version 7.5). The solid samples and only liquid Zr(OPr)_4_ were measured directly, without sample preparation, using the attenuated total reflectance module (Platinum ATR). Inductively coupled plasma optical emission spectrometry (ICP-OES) was measured on a PerkinElmer optical emission spectrometry Optima 8300 instrument.

### Synthesis of Clusters as Reported in the Literature

#### [Zr_6_(OH)_4_O_4_(OOCCH_3_)_12_]_2_ (**3**)^24^

In a Schlenk tube under a nitrogen atmosphere, Zr(O^*n*^Bu)_4_ (2.13 g of an 80% wt % solution in *n*-butanol, 4.44 mmol) was mixed with acetic acid (2.64 g, 44.0 mmol) in dry CH_2_Cl_2_ (5 mL). After homogenization for a few minutes, the mixture was kept at room temperature. A white solid precipitated within 3 h. It was collected by centrifugation and dried under high vacuum at room temperature (yield: 0.66 g, 52% based on Zr, and considering the reported chemical formula [Zr_6_(OH)_4_O_4_(OOCCH_3_)_12_]_2_·6CH_3_COOH·3.5CH_2_Cl_2_). Spectroscopic data agreed with previous reports.^24,25^

#### [Zr_6_O_4_(OH)_4_(OOCCH_2_CH_3_)_12_]_2_ (**6**)^24^

In a Schlenk tube under a nitrogen atmosphere containing a magnetic stirring bar and Zr(OnBu)_4_ (1.08 mL of an 80% wt % solution in *n*-butanol, 2.37 mmol), propionic acid (1.80 mL, 24.2 mmol) was added dropwise. After 7–8 h at room temperature, colorless crystals were formed. The crystals were separated and dried in vacuo (0.26 g, 37% based on Zr, and considering the reported formula [Zr_6_O_4_(OH)_4_(OOCCH_2_CH_3_)_12_]_2_·6CH_3_CH_2_COOH). Spectroscopic data agreed with previous reports.^24,25^

#### [Zr_6_(OH)_4_O_4_(OAcr)_12_]_2_·6AcrOH (OAcr = Acrylate) (**7**)^29^

This cluster was prepared as described in our previous study.^22^

#### [Zr_6_O_4_(OH)_4_(C_2_H_3_O_2_)_8_(H_2_O)_2_Cl_3_] (**8**)^32^

In a round-bottom flask equipped with a reflux condenser and a magnetic stirring bar, ZrCl_4_ (2.0 g, 8.4 mmol) was added to a mixture of 3 mL of acetic acid and 5 mL of isopropanol under stirring. The mixture was heated at 120 °C for 60 min resulting in the formation of a white solid. The white solid product was collected by centrifugation (30 min at 5000 rpm). The collected white solid was subsequently washed with acetone (2 × 35 mL) and dried under vacuum at room temperature. Yield: 1.71 g, 95% based on Zr, and considering the reported formula [Zr_6_O_4_(OH)_4_(C_2_H_3_O_2_)_8_(H_2_O)_2_Cl_3_].^32^

#### [Zr_6_O_4_(OH)_4_(OMc)_12_] (OMc = Methacrylate) (**12**)^35,36,50^

This cluster was prepared as described in our previous study.^23^

### Cluster Formation under Amidation Conditions

#### [Zr_6_(OH)_4_O_4_(OOCCH_3_)_12_]_2_ (**3**)

A 4 mL vial was charged with Zr(O^*n*^Pr)_4_ (0.27 mL of a 70% wt % solution in *n*-propanol, 0.60 mmol), acetic acid (10 mmol, 0.57 mL), solvent (3.0 mL of EtOH, MeCN, toluene, or 1,4-dioxane), and magnetic stirring bar. The mixture was stirred at 80 °C for 1 h. A solid precipitated from the solution after a few minutes. After 1 h, the solid was collected through centrifugation and then dried under vacuum at room temperature. Yield (in EtOH): 0.16 g, >99% based on Zr, and considering the formula [Zr_6_(OH)_4_O_4_(OOCCH_3_)_12_]_2_·6CH_3_COOH.

#### [Zr_6_O_4_(OH)_4_(OOCCH_2_CH_3_)_12_]_2_ (**6**)

A 4 mL vial was charged with Zr(O^*n*^Pr)_4_ (0.27 mL of a 70% wt % solution in *n*-propanol, 0.60 mmol), propionic acid (10 mmol, 0.75 mL), EtOH (3 mL), and magnetic stirring bar. The mixture was stirred at 80 °C for 1 h. After 1 h, the solvent was evaporated under reduced pressure. The solid obtained was washed with 2 mL of MeCN and then dried under vacuum at room temperature. Yield: 0.18 g, >99% based on Zr, and considering the reported formula [Zr_6_O_4_(OH)_4_(OOCCH_2_CH_3_)_12_]_2_·6CH_3_CH_2_COOH. For ^1^H NMR, the solid (∼10 mg) was solubilized in 500 μL of CD_2_Cl_2_. The final solution was centrifuged with the supernatant transferred to an NMR tube, and ^1^H NMR was recorded.

#### [Zr_6_(OH)_4_O_4_(OAcr)_12_]_2_ (OAcr = Acrylate) (**7**)

A 4 mL vial was charged with Zr(O^*n*^Pr)_4_ (0.27 mL of a 70% wt % solution in *n*-propanol, 0.60 mmol), acrylic acid (10 mmol, 0.69 mL), EtOH (3 mL), and a magnetic stirring bar. The mixture was stirred at 80 °C for 1 h. A solid precipitated from the solution after a few minutes. After 1 h, the solid was collected through centrifugation and then dried under vacuum at room temperature. The same procedure was used when the solvent used was 1,4-dioxane. Yield (in EtOH): 0.18 g, >99% based on Zr, and considering the formula [Zr_6_(OH)_4_O_4_(OAcr)_12_]_2_·6AcrOH. For ^1^H NMR, the solid (∼10 mg) was solubilized in 500 μL of DMSO-*d*_6_. The final solution was centrifuged, the supernatant was transferred to an NMR tube, and ^1^H NMR was recorded.

#### [Zr_6_O_4_(OH)_4_(OMc)_12_] (OMc = Methacrylate) (**12**)

A 4 mL vial was charged with Zr(O^*n*^Pr)_4_ (0.27 mL of a 70% wt % solution in *n*-propanol, 0.60 mmol), methacrylic acid (10 mmol, 0.85 mL), EtOH (3 mL), and magnetic stirring bar. The mixture was stirred at 80 °C for 1 h. A solid precipitated from the solution after a few minutes. After 1 h, the reaction mixture was evaporated to afford an oil that contained a white solid. The oil was carefully removed using a Pasteur pipet, and the solid was washed 3 times by dissolving the mixture in 15 mL of CHCl_3_ and evaporating under reduced pressure. The white solid obtained was then dried under a vacuum at room temperature. Yield: 0.16 g, 92% based on Zr, and considering the formula [Zr_6_(OH)_4_O_4_(OMc)_12_]. For ^1^H NMR, the solid (∼10 mg) was solubilized in 500 μL of CDCl_3_. The final solution was centrifuged, the supernatant was transferred to an NMR tube, and ^1^H NMR was recorded.

#### Alternative Zr Salts (Zr(OBu)_4_, ZrCl_4_, ZrOCl_2_) for Cluster Formation under Amidation Conditions

Similar procedures were used for alternative Zr salts; details are given in the Supporting Information.

### Procedures for Amide Formation in Table 1

#### Method A

A 4 mL vial was charged with Zr(OPr)_4_ (0.03 mmol, 14 μL), acetic acid (0.50 mmol, 28.0 μL), ethanol (0.15 mL), benzylamine (161 mg, 1.50 mmol), and magnetic stirring bar. The mixture was stirred overnight at 80 °C. For ^1^H NMR, the reaction mixture was diluted with CDCl_3_ (1 mL), 3,5-bistrifluoromethyl-bromobenzene (1.0 equiv) was added as an internal standard, and the reaction mixture was stirred at room temperature for 10 min to ensure thorough mixing. Next, 50 μL of the crude mixture was transferred to a 1.5 mL centrifuge tube and diluted with 450 μL of CDCl_3_. The final solution was centrifuged. The supernatant (∼500 μL) was transferred to an NMR tube, and ^1^H NMR was recorded. Results are reported based on ^1^H NMR yields.

#### Method B

A 4 mL vial was charged with Zr(OPr)_4_ (0.03 mmol, 14 μL), acetic acid (0.50 mmol, 28.0 μL), ethanol (0.15 mL), and a magnetic stirring bar. The mixture was stirred at 80 °C for around 1 h. Then benzylamine (161 mg, 1.50 mmol) was added, and the solution was stirred overnight at 80 °C. For ^1^H NMR, the reaction mixture was diluted with CDCl_3_ (1 mL), 3,5-bistrifluoromethyl-bromobenzene (1.0 equiv) was added as an internal standard, and the reaction mixture was stirred at room temperature for 10 min to ensure thorough mixing. Next, 50 μL of the crude mixture was transferred to a 1.5 mL centrifuge tube and diluted with 450 μL of CDCl_3_. The final solution was centrifuged. The supernatant (∼500 μL) was transferred to an NMR tube and ^1^H NMR was recorded. Results are reported based on ^1^H NMR yields. The same procedure was used for the gram-scale reaction in Table 1 (entry 9).

### Extended X-Ray Absorption Fine Structure (EXAFS)

XAFS experiments were performed at the XAFS 11.1 beamline of Elettra Sincrotrone Trieste (Italy).^51^ The storage ring operated at 2.4 GeV in the top-up mode with a typical current of 310 mA. XAFS data were recorded at the Zr K-edge in transmission mode using either pellets made upon mixing a proper amount of ex situ samples (Table S1) and cellulose or the in situ setup. Spectra were acquired from 17698 to 19232 eV around the Zr K-edge with a constant k-step of 0.03 Å^–1^ and 1 s/point acquisition time.

As shown in Figure S9a, the in situ setup consisted of four main elements: (i) a 2 mL Eppendorf tube in polypropylene as the reaction vessel, tightly fitted in (ii) an aluminum heating block with windows to allow X-rays to pass through the sample; (iii) a temperature probe inserted in the heating block and connected to (iv) a heating plate that was used to maintain the reaction temperature at 76 °C. The reaction studied consisted of 475 mg of phenylacetic acid (**11**) added to a solution of Zr_12_ cluster **7** (122 mg) in EtOH (1.1 mL). This initial mixture was homogenized manually and heated at 80 °C for approximately 1 h to acquire the first two XAFS spectra of the in situ data set. Then, 1.1 mL of benzylamine (**9**) was added, and the heating continued for another 16 h (addition occurred in correspondence to the third spectrum). Continuous XAFS spectra acquisition occurred for about 17 h in total, recording one spectrum every ∼23 min (Table S2).

The extended X-ray absorption fine structure (EXAFS) analysis was performed using the GNXAS package^52,53^ based on the multiple scattering (MS) theory. The sinusoidal signal of the experimental EXAFS spectra was described by considering the model in Figure S9b. Each Zr atom (for instance, the dark blue one) is in an 8-fold coordination environment, including four μ_3_O and four μ_2_O atoms. Only a few key contributions were taken into account in describing the experimental EXAFS signal. Specifically, (i) two two-body (γ^(2)^) term signals were used to account for the two different structural oxygens in the Zr first-shell environment (i.e., Zr-μ_3_O and Zr-μ_2_O), and (ii) an additional γ^(2)^ signal was used to describe the Zr–Zr contribution in the second-shell sphere (degeneracy of four, interatomic distance ∼3.6 Å). The inclusion of an additional Zr–Zr contribution for the opposite pair (degeneracy of one, interatomic distance ∼5 Å) did not produce a significant improvement in the fit.

### Computational Section

#### DFT Calculations

DFT calculations were performed at the B3LYP level^54−57^ using Gaussian16, revision A03 software.^58^ Geometry optimizations were carried out using the LANL2DZ^59^ basis set and associated pseudopotentials for Zr atoms and the Pople-type 6-31G basis set^60−62^ for the remaining ones (C, H, O, and N). Electronic energies were corrected via single-point calculations on the optimized geometries using a more extended triple-ξ basis set consisting of a LANL2TZ basis set^63^ supplemented with f-type polarization functions^64^ for Zr and 6- 311++G(2d,2p)^65−67^ for remaining atoms. Solvent effects of DMSO were included in all of the calculations using SMD model^68^ as implemented in Gaussian16.^58^ The stationary-point nature of both minima and transition-state structures has been confirmed by frequency calculation analysis. To better account for weak dispersion interactions, we employed the Grimme’s D3 empirical dispersion correction using a Becke–Johnson finite damping (D3BJ).^69−71^ Quasi-harmonic correction was applied to frequencies below 100 cm^–1^ by means of the Goodvibes code.^72^ Gibbs free energies were calculated at the standard state of 1 mol·L^–1^ and 25 °C and were scaled to experimental temperatures using the Goodvibes code.^72^ A data set of the most representative structures from the reported mechanisms is available in the ioChem-BD repository^73^ and can be accessed via the following link: 10.19061/iochem-bd-2-64.

#### Molecular Dynamics (MD) Simulations

Molecular dynamics (MD) simulations were carried out using the GROMACS 5.1.2 code^74−77^ and AMBER99 force field,^78^ following the methodology that was successfully applied for evaluating the interactions between metal oxo clusters and amino acids.^41,79^ The potential energy of system U is empirically described as the sum of bonding terms, including bond, angle, and dihedral deformation energies, and nonbonding terms, which consist of pairwise additive 1–6–12 electrostatic and van der Waals potentials. The latter is employed to describe interactions between atoms in different molecules or those separated by more than three bonds within the same molecule.

The core of the Zr oxo cluster (**12**) has been treated as a semirigid body, with force constants of 900 kJ mol^–1^ nm^–2^ and 900 kJ mol^–1^ rad^–2^ for bonds and angles, respectively, whereas the ligands were described by AMBER99 parameters. Atomic charges were obtained following the procedure outlined by Bonet-Ávalos et al.,^80,81^ whereby a single-point calculation is performed on the optimized structure of **12** to compute the CHELPG charges derived from the electrostatic potential, using the same theory level described above. Lennard-Jones parameters for Zr, O, and H atoms of the Zr oxo core were obtained from previous works.^82^ To model the junction between the rigid cluster and the flexible carboxylates of the ligands, an additional parameter reported by Yang et al. for describing UiO-66 MOFs^83^ was used to describe the Zr–O_ligand_–C1_ligand_–C2_ligand_ torsion with a force constant of 86.837 kJ mol^–1^ rad^–2^.

DMSO was employed as the solvent in the simulations, which was modeled with the force field developed by van der Spoel et al.^84^ Water molecules were represented using the TIP3P model.^85^ All simulations were performed under three-dimensional (3D) periodic boundary conditions in a cubic box of 63^3^ Å^3^ using an interatomic distance cutoff of 14 Å for van der Waals and Coulombic interactions. Long-range electrostatics were corrected using the particle–particle mesh Ewald (PME) summation method.^86^

All simulations were performed within a canonical ensemble (NVT), starting from randomly distributed initial velocities at 343 K. Newton equations of motion were integrated using the Verlet algorithm^87^ with a time step of 1 fs. All along the runs, the temperature was controlled by the Bussi–Donadio–Parrinello thermostat, which relies on a velocity rescaling algorithm that ensures a canonical distribution for kinetic energy.^88^ A relaxation time of 0.1 ps was used for all particles, except for the Zr oxo cluster. For the latter, a relaxation time of 0.02 ps was used to prevent sudden variations in the temperature due to the semirigid nature of the cluster. For equilibrations within the isothermal–isobaric (NPT) ensemble, the system was coupled to the Parrinello–Rahman barostat^89^ at 1 bar. Before the production runs, all systems underwent energy minimization followed by an equilibration protocol that consists of a 500 ps run at constant volume and temperature (NVT ensemble) and 500 ps at constant pressure to adjust the box dimensions and, intrinsically, the density. Finally, 20 ns of production simulations were conducted within an NVT ensemble collecting data every 1 ps from the trajectories.

## Supporting Information Available

Experimental procedures; supporting experiments; copy of NMR spectra; computational details; and supporting computational results (PDF)
