# Supplementary material for: Mechanism Insight into Direct Amidation Catalyzed by Zr Salts: Evidence of Zr Oxo Clusters as Active Species
Source: Inorg Chem. 2024 Oct 18;63(43):20347–60. doi: 10.1021/acs.inorgchem.4c02526 (PMC11590061; doi:10.1021/acs.inorgchem.4c02526)
Supplement: Supplementary file 1 — ic4c02526_si_001.pdf [file ic4c02526_si_001.pdf]

Supporting Information

Mechanism Insight into Direct Amidation Catalyzed by Zr Salts: Evidence of Zr Oxo Clusters as Active Species

Yujie Zhang[a,c,†], Jordi Puiggalí-Jou [b,†], Angelo Mullaliu[a,†], Albert Solé-Daura [b], Jorge J. Carbó [b,*], Tatjana N. Parac-Vogt [a,*] and Francisco de Azambuja [a,*]

[a] Department of Chemistry, KU Leuven, Celestijnenlaan 200F, 3001 Leuven, Belgium

[b] Department de Química Física i Inorgànica, Universitat Rovira i Virgili, Tarragona 43007, Spain

[c] Present address: College of Chemistry and Environmental Engineering, Wuhan Polytechnic University, Wuhan 430023, China

* francisco.deazambuja@kuleuven.be; tatjana.vogt@kuleuven.be; j.carbo@urv.cat

† Authors contributed equally to this work

Table of contents

Formation of Zr Oxo Clusters In-situ....2

Cluster formation under amidation conditions....2

Alternative Zr salts for cluster formation under amidation conditions....6

Extended X-ray Absorption Fine Structure (EXAFS)....8

General remarks....8

EXAFS supplementary results....10

Computational Study....13

Formation and decomposition analysis of Zr_n oxo clusters....13

Design of a suitable computational model....16

Mechanistic investigation....17

Molecular Dynamics study of ZrOCs clusters in solution: water and DMSO solvent mixtures....20

References....24

Formation of Zr Oxo Clusters In-situ

Cluster formation under amidation conditions

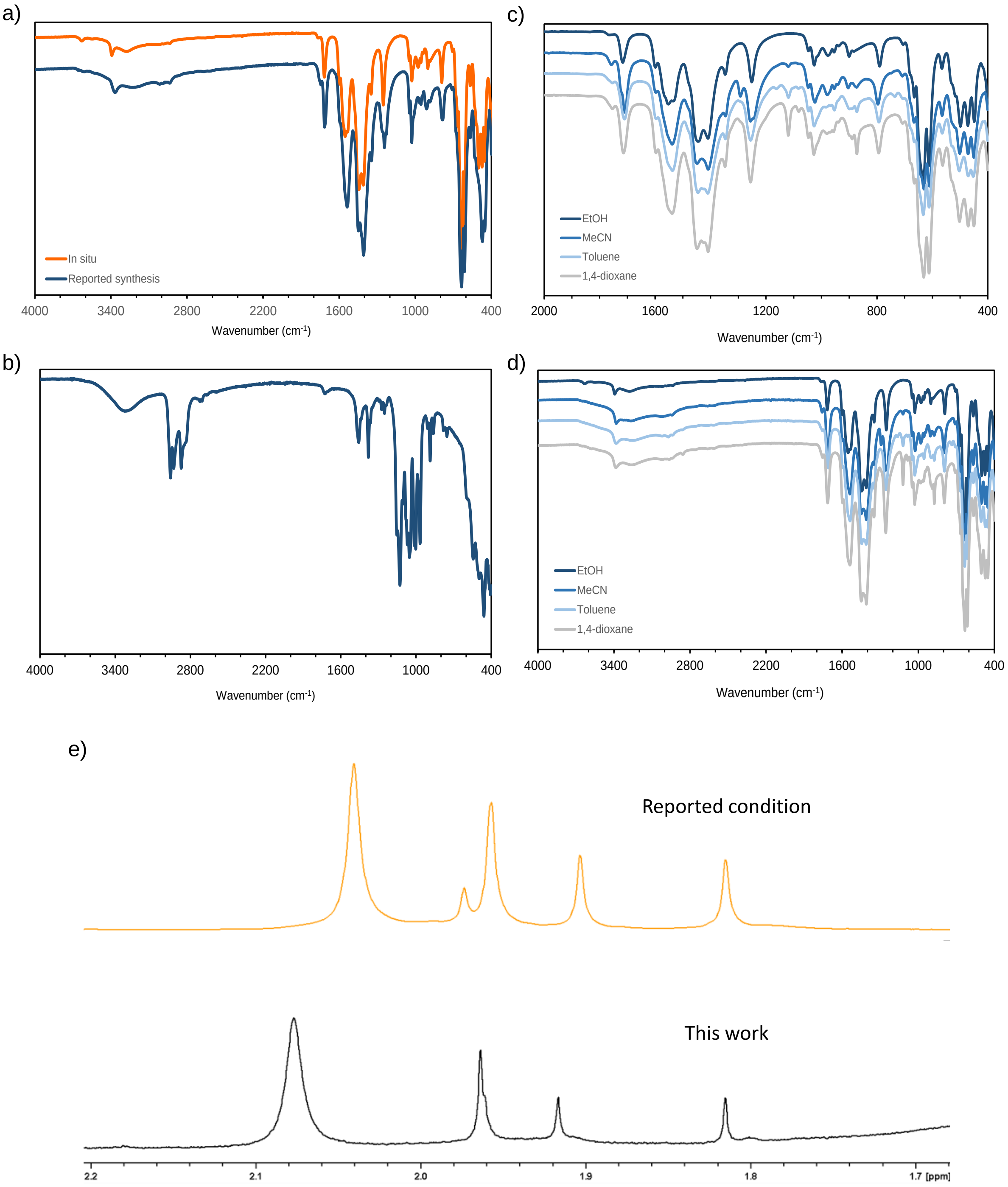

Figure S1. a) Comparison of full IR spectra of cluster $[Zr_6(OH)_4O_4(OOCCH_3)_{12}]_2$ (**3**) formed *in situ* as described above with a batch obtained following previous literature reports (*reported synthesis*) (ref. [1-2]). b) IR analysis of $Zr(OPr)_4$. c) IR analysis of different batches of **3** obtained from a mixture of acetic acid and $Zr(OPr)_4$ in different solvents. d) Full IR spectra of the magnifications shown in 'c'. e) ^1H NMR spectra ($CDCl_3$) of $[Zr_6O_4(OH)_4(OOCCH_3)_{12}]_2$ (**6**) synthesized in-situ in EtOH (bottom), and using previously reported conditions (top).

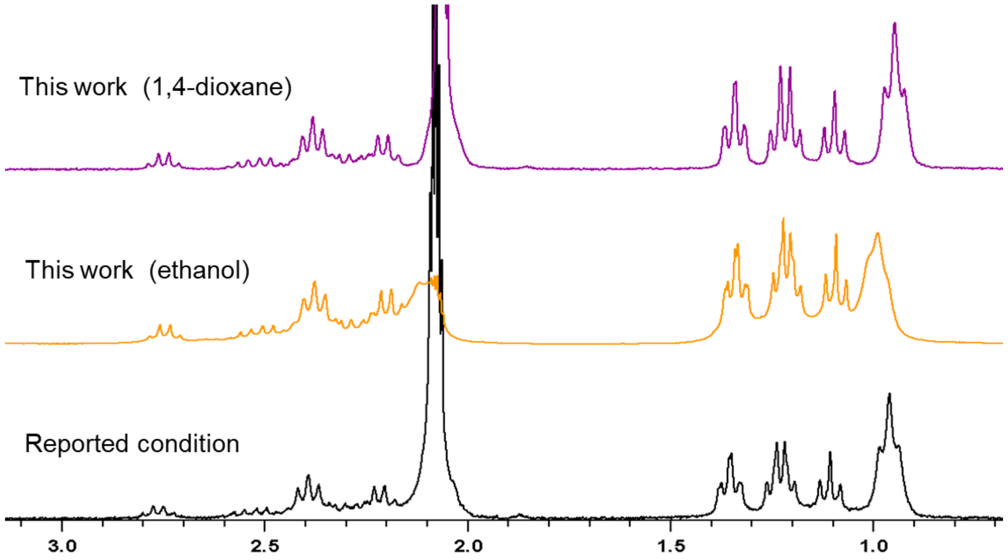

Figure S2. ^{1}H NMR spectra (toluene-d$_8$) of $[Zr_6O_4(OH)_4(OOCCH_2Me)_{12}]_2$ (**6**) synthesized in-situ in 1,4-dioxane (top) or ethanol (center), and using previously reported conditions (bottom).

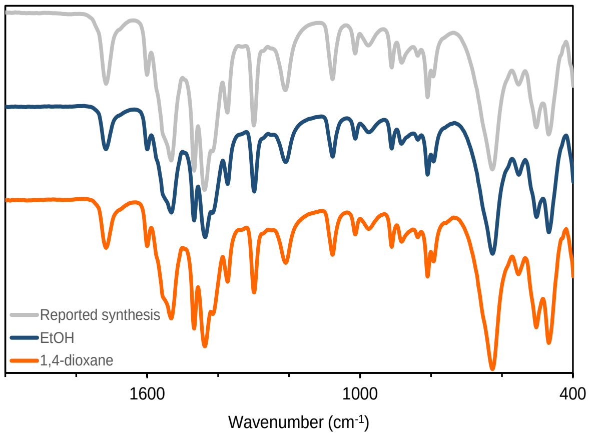

Figure S3. IR analysis of $[Zr_6O_4(OH)_4(OOCCH_2Me)_{12}]_2$ (**6**) cluster obtained *in situ* from a mixture of propionic acid (**4**) and $Zr(OPr)_4$ in ethanol, and in 1,4-dioxane.

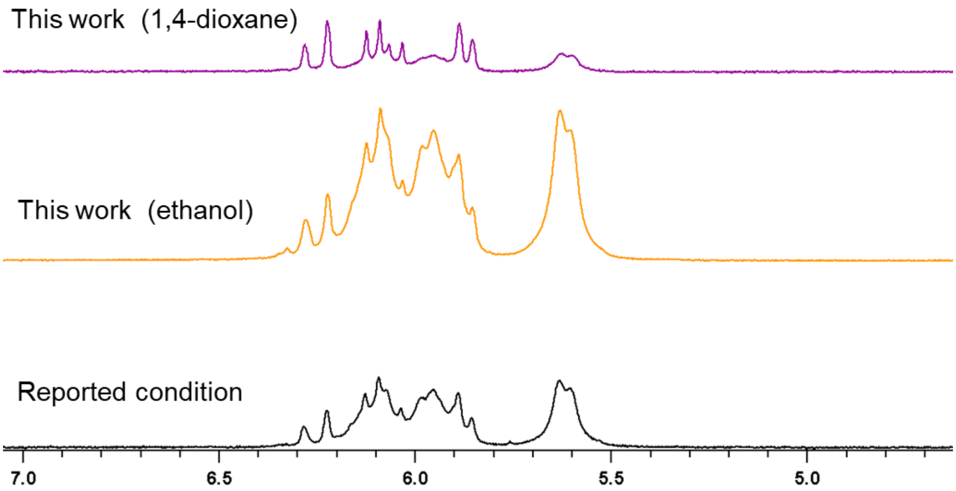

Figure S4. ^{1}H NMR spectra (DMSO-d_6) of [$Zr_6O_4(OH)_4(OAcr)_{12}$] (OAcr = acrylate) (**7**) synthesized in-situ in 1,4-dioxane (top) or ethanol (center), and using previously reported conditions (bottom).

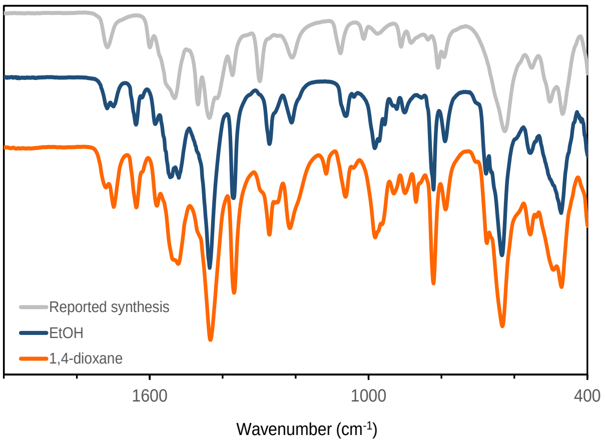

Figure S5. IR analysis of [$Zr_6O_4(OH)_4(OAcr)_{12}$] (OAcr = acrylate) (**7**) cluster obtained *in situ* from a mixture of acrylic acid (**5**) and $Zr(OPr)_4$ in ethanol, and in 1,4-dioxane.

a)

b)

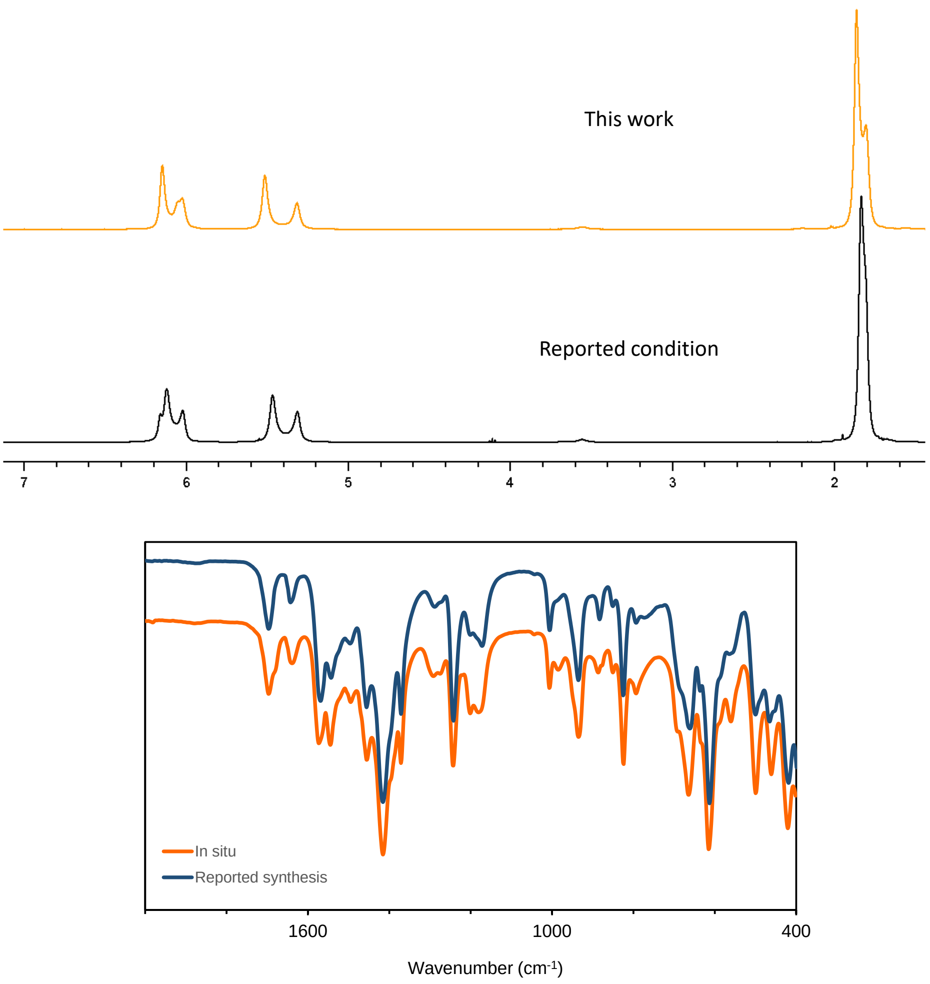

Figure S6. a) ^{1}H NMR spectra ($CDCl_3$) of [$Zr_6O_4(OH)_4(OMc)_{12}$] (OMc = methacrylate) (**12**) synthesized in-situ in ethanol (top) and using previously reported conditions (bottom). b) IR analysis [$Zr_6O_4(OH)_4(OMc)_{12}$] (OMc = methacrylate) (**12**) synthesized in-situ in ethanol and using previously reported conditions.

Alternative Zr salts for cluster formation under amidation conditions

Zr(OBu)$_4$:

A 4 mL vial was charged with Zr(OBu)$_4$ (0.03mmol, 14 µL), acetic acid (0.50 mmol, 28.0 µL), solvent (0.15 mL), and magnetic stirring bar. The mixture was stirred at 80 °C for around 1 hour. There was solid that precipitated from the solution after a few minutes. The solid was collected through centrifugation and then dried under vacuum oven.

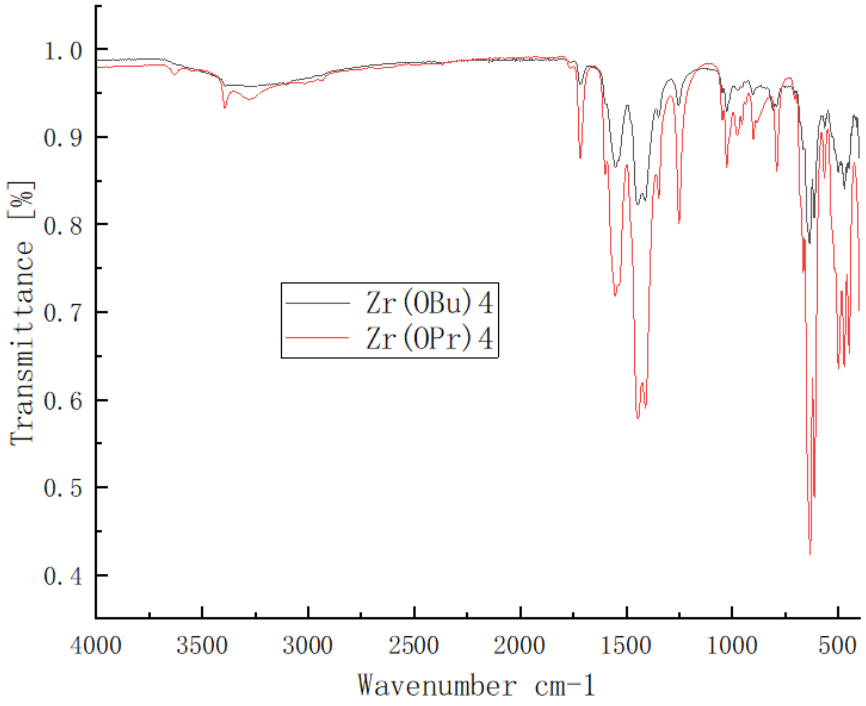

Figure S7. IR of $[Zr_6(OH)_4O_4(OOCCH_3)_{12}]_2$ (**3**) obtained using different zirconium alkoxides.

ZrCl$_4$ / ZrOCl$_2$:

As discussed in the main text, chlorinated Zr source such as $ZrCl_4$ and $ZrOCl_2$ afforded different Zr oxo clusters. Based on previous literature, we hypothesized the cluster formed could be $[Zr_6O_4(OH)_4(C_2H_3O_2)_8(H_2O)_2Cl_3]$ (**8**) since formation of Zr_6 cluster **8** using $ZrCl_4$ as precursor has been reported by mixing it with acetic acid at 120°C for one hour in propanol.[3] Consequently, it is reasonable to expect formation **8** under amidations conditions. To show that, we have gradually modified the reported the condition to make sure the same hexanuclear ZrOCs formation was obtained. The following experiments were tried:

1) First, the literature condition was repeated decreasing the temperature from 120 °C to 80 °C – specifically, $ZrCl_4$ (2.0 g, 8.5 mmol) was added into a mixture of 3 mL of acetic acid and 5 mL of isopropanol under stirring at 500 rpm and heated at 80 °C for 60 min. The product was collected either through suction filtration or centrifugation at 10,000 rpm. The collected white solid was subsequently washed with acetone twice and dried under vacuum at room temperature.

2) In a second attempt, the solvent was changed from propanol to ethanol, and the concentration of acetic acid was maintained in 10.5 mol L^{-1}. Meanwhile, the concentration of $ZrCl_4$ was decreased from 1.7 mol L^{-1} to 0.6 mol L^{-1}, so that the ratio acid/Zr present in our amidation reactions was reproduced – specifically, a 4 mL vial was charged with $ZrCl_4$ (0.14 g, 0.60 mmol), acetic acid (10 mmol, 0.57 mL), EtOH (0.95 mL), and magnetic stirring bar. The mixture was stirred at 80 °C for 1 hour. The compound was collected through evaporation of the solvent, then dried under vacuum at 50 °C in a vacuum oven. Yield (in EtOH): 0.10 g, 77% based on Zr and considering the formula $[Zr_6O_4(OH)_4(C_2H_3O_2)_8(H_2O)_2Cl_3]$.

3) Finally, the concentrations of both $ZrCl_4$ and acetic acid were adjusted to mimic the condition of the amidation reactions. The same procedure as described in 2 was followed, but using 3.0 mL of ethanol was used as solvent instead.

The IR and far-IR spectrum of all the compounds obtained match well with the one from cluster **8** prepared according to the literature, indicating **8** is likely the cluster being formed in-situ when $ZrCl_4$ is used as the Zr precursor (**Figure S8**).

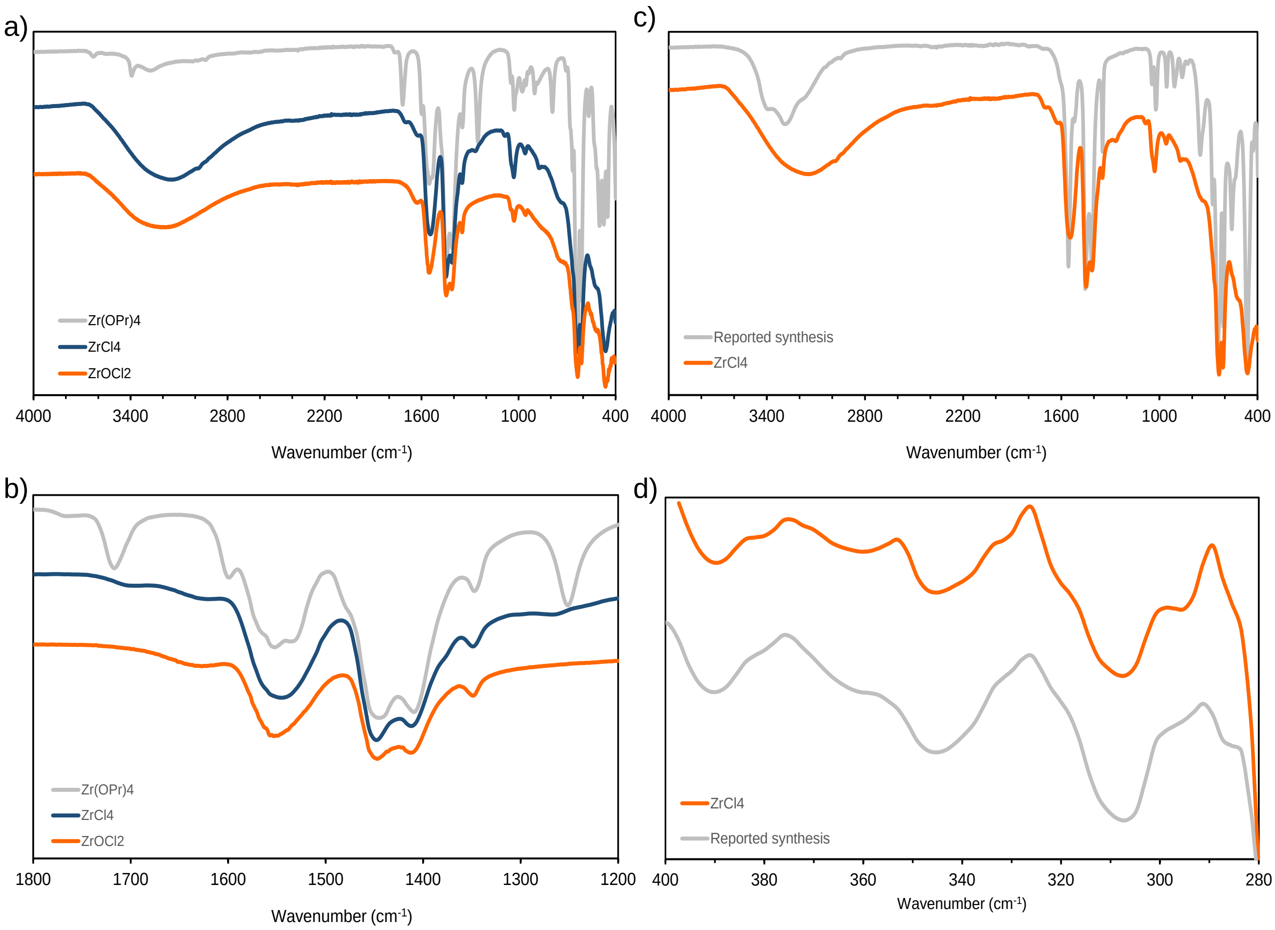

Figure S8. Chlorinated Zr salts ($ZrCl_4$, and $ZrOCl_2$) afforded hexanuclear ZrOC $[Zr_6O_4(OH)_4(C_2H_3O_2)_8(H_2O)_2Cl_3]$ (**8**) under the same conditions in which Zr alkoxides provided dodecanuclear cluster **3**. a) IR spectra of the solids obtained using different Zr salts; b) Magnification of IR spectra presented in 'a'. c) IR spectra of hexanuclear ZrOC $[Zr_6O_4(OH)_4(C_2H_3O_2)_8(H_2O)_2Cl_3]$ (**8**) formed using literature condition and condition in this work. d) Far IR spectra of **8** formed using literature condition and condition in this work.

Extended X-ray Absorption Fine Structure (EXAFS)

General remarks

(a) (b)

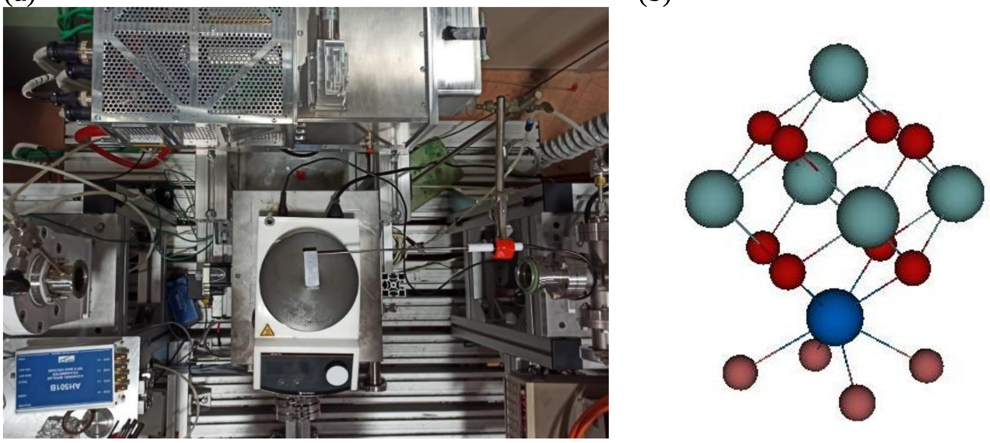

Figure S9. a) Experimental set-up for the reaction in-situ; b) graphical representation of the EXAFS structural model (Zr atoms: blue/ light blue; μ_3O atoms: red; μ_2O atoms: salmon). *μ_3O atoms* = Zr-μ_3O(H) bonds (inorganic [Zr_6O_8] core); *μ_2O atoms* = Zr-O/N_{ligand} bonds.

Table S1. Labels and description of ex-situ samples analyzed by EXAFS

Sample code	Sample description	Sample code	Sample description
FD-031-1	Zr_{12}-OAcrylate (Zr_{12}-OAcr)	FD-031-2	Zr_6-OMethacrylate (Zr_6-OMc)
FD-031-3	Zr_{12}-OAcr + benzylamine	FD-031-4	Zr_6-OMc + benzylamine
FD-031-5	Zr_{12}-OAcr + phenylacetic acid	FD-031-6	Zr_6-OMc + phenylacetic acid
FD-031-7	Zr_{12}-OAcr + phenylacetic acid: benzylamine 1:2	FD-031-8	Zr_6-OMc + phenylacetic acid: benzylamine 1:2

Table S2. Correlation between XAFS spectrum number and reaction time for the formation of amide **14** followed *in situ*.

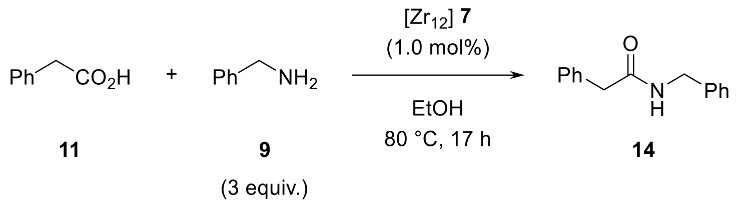

Spectrum	Time / h	Spectrum	Time / h	Spectrum	Time / h	Spectrum	Time / h
0	0.38	11	4.60	22	8.82	33	13.03
1	0.77	12	4.98	23	9.20	34	13.42
2	1.15	13	5.37	24	9.58	35	13.80
3	1.53	14	5.75	25	9.97	36	14.18
4	1.92	15	6.13	26	10.35	37	14.57
5	2.30	16	6.52	27	10.73	38	14.95
6	2.68	17	6.90	28	11.12	39	15.33
7	3.07	18	7.28	29	11.50	40	15.72
8	3.45	19	7.67	30	11.88	41	16.10
9	3.83	20	8.05	31	12.27	42	16.48
10	4.22	21	8.43	32	12.65	43	16.87
						44	17.25

EXAFS supplementary results

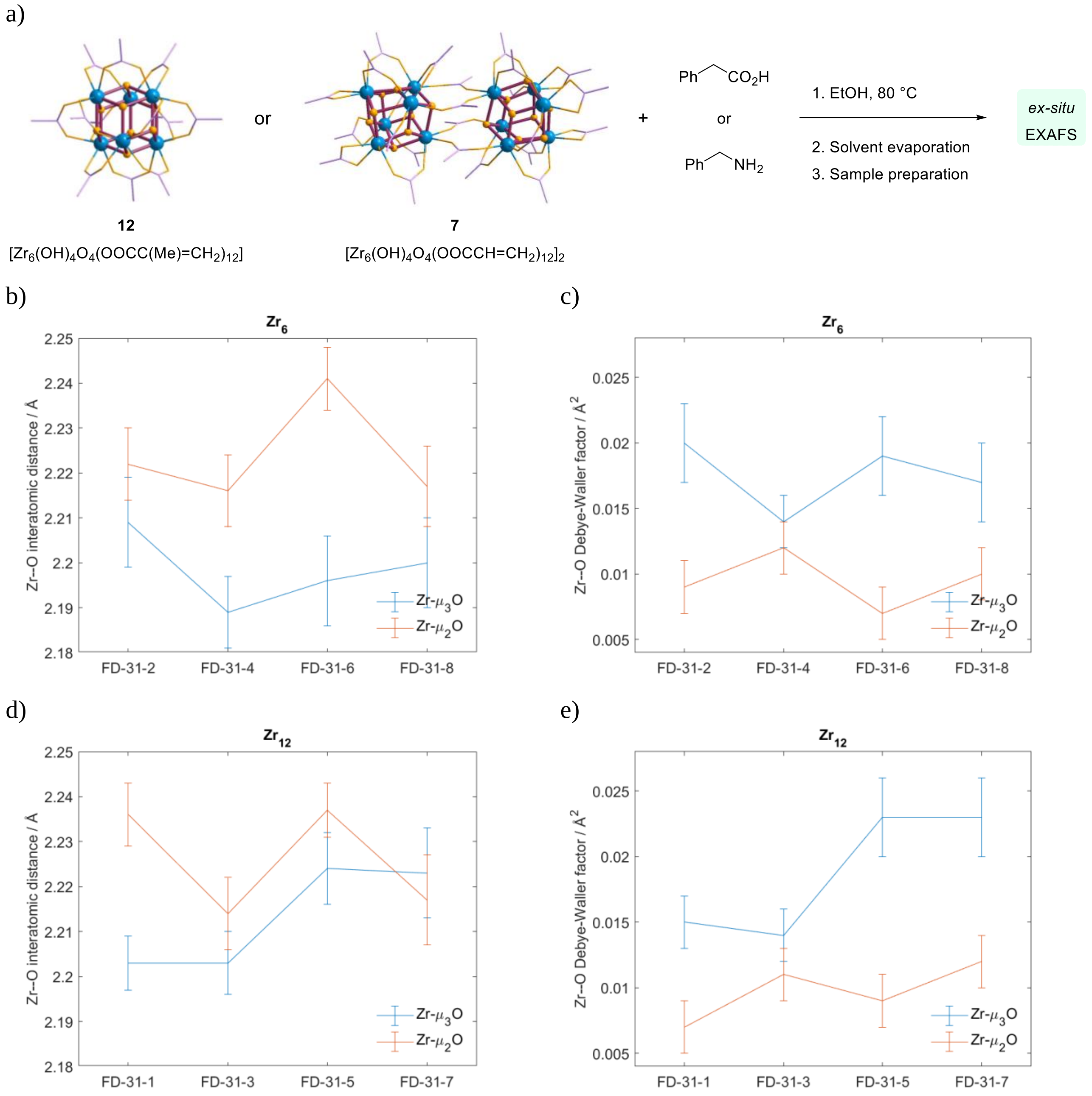

Figure S10. EXAFS results of samples analyzed ex-situ (see **Table S1**). a) Schematic representation of samples used in ex-site EXAFS analysis. b)Variation in Zr-O bond lengths and c) Debye-Waller factors for Zr_6 cluster **12**. d) Variation in Zr-O bond lengths and e) Debye-Waller factors for Zr_{12} cluster **7**. *Legend:* Zr-μ_3O refers to Zr-O bonds of the inorganic [Zr_6O_8] core; Zr-μ_2O refer to Zr-capping ligand interactions. When amine is present, the Zr-O_{COOH} fragment accounts for both Zr-O and Zr-N bonds as backscattering amplitude of consecutive light elements results in negligible differences.

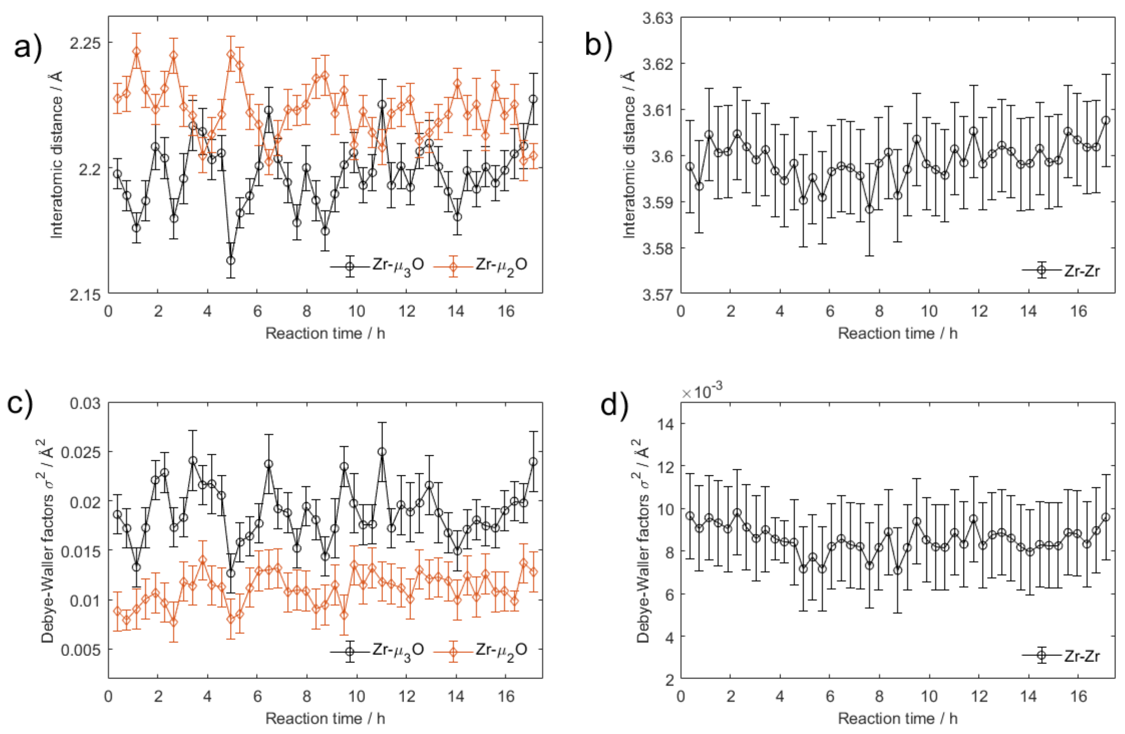

Figure S11. *In situ* EXAFS analysis of the amidation reaction between phenylacetic acid (**11**) and benzylamine (**9**) catalyzed by Zr_{12} cluster **7** for 17 hours (**Table S2**): a) Variations of Zr-μ_2O and Zr-μ_3O bond lengths and c) respective trend in Debye-Waller factors as a result of structural disorder. b) Variation of intra-cluster Zr-Zr distances and d) respective Debye-Waller factors. Spectra 1 and 2 consist of a mixture of **7** and **11**, while addition of **9** occurred in correspondence of spectrum 3.

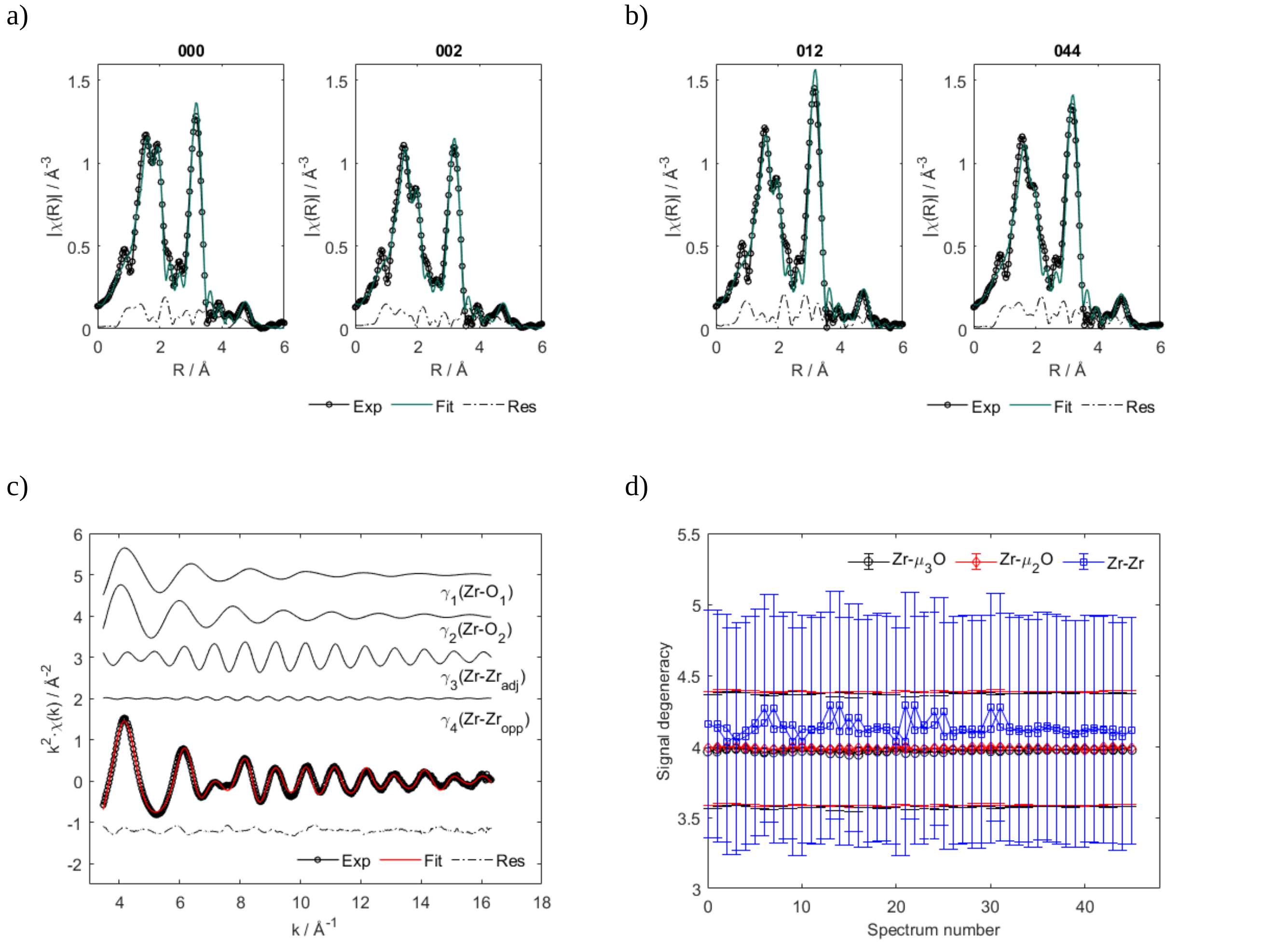

Figure S12. EXAFS fitting results for the in-situ dataset. a-b) Representative examples of fit for a few relevant datapoints. c) Fit on spectrum 0 is presented in k-space together with the two-body scattering signals considered to describe the experimental EXAFS spectrum. d) Trend in signal degeneracy (i.e., coordination number) for the three main scattering contributions taken into consideration during analysis.

Panels of **Figure S12** show representative examples of fit for a few relevant datapoints. The fits in a) and b) are presented in the R-space for the spectra 0, 2, 12, and 44, highlighting the general very good match between experimental and calculated signals. In panel c), the fit of calculated signals on spectrum 0 is presented in k-space together with the two-body scattering signals considered to describe the experimental EXAFS spectrum. Here, it is clear that the fourth two-body term $\gamma_4^{(2)}$ arising from opposite/apical Zr atoms has a low amplitude and could be neglected from the final fit. Finally, panel d) shows a stable trend in signal degeneracy (i.e., coordination number) for the main three scattering contributions taken into consideration during analysis. In particular, a constant degeneracy value for the Zr--Zr fragment strongly indicates a stable structural conformation of the [Zr_6O_8] cluster core during the entire duration of the reaction.

Computational Study

Formation and decomposition analysis of Zr_n oxo clusters

We have performed calculations on the thermodynamics of various Zr oxo clusters with different nuclearity (Zr_2, Zr_3, Zr_4, and Zr_5), exploring their energy landscape in the presence of carboxylic acids. Naturally, macroscopic and kinetic effects may also govern the reaction kinetics towards an observed nuclearity; however, as this is beyond the scope of this work, we sought to gauge the thermodynamic stability of different clusters under the conditions of our work. Our analysis considered two distinct processes: i) the formation free energies for Zr_2, Zr_3, Zr_4, Zr_5, and Zr_6 oxo clusters starting from methacrylic acid and the two different Zr salts used experimentally, $Zr(OPr)_4$ and $ZrCl_4$ (**Table S3**); and ii) the decomposition of Zr_6 cluster **12** through different pathways yielding Zr_1 + Zr_5, Zr_2 + Zr_4, or Zr_3 + Zr_3 fragments (**Table S4**). In our calculations, we considered that degradation could occur through hydrolysis and alcoholysis reactions, and solvent molecules would coordinate to the resulting species ensuring a 7-fold coordination of Zr centers. Accordantly, the systematic study of coordination patterns for monomeric Zr species evidenced that 7-coordinated Zr is the most stable species (see **Figure S13**). The molecular models of potential Zr_n (n = 2 – 6) oxo clusters were constructed adapting reported X-ray[4-5] or DFT[6] structures as references, even though each Zr_n cluster has been prepared using different experimental conditions to direct the formation of a given species.

All the computed energies further support the preponderant role of Zr_6 oxo cluster under current experimental conditions, as aslo evidenced by our mechanistic studies. As shown in **Table S3**, the formation of Zr_6 clusters is the most favorable process in DMSO, starting with either $Zr(OPr)_4$ or $ZrCl_4$ salts. Furthermore, all the computed decomposition free energies (see **Table S4**) for all the explored pathways were large enough (> 38 kcal mol^{-1}) to support the stability of Zr_6 oxo cluster under current experimental conditions. Therefore, the formation of Zr_6 oxo clusters in situ is feasible, and results in a stable species that can catalyze amide bond formation, completely in line with our thorough mechanistic study reported in the manuscript.

Table S3. Reaction free-energies (kcal mol^{-1}) for the formation of Zr_n oxo clusters (n = 3 – 6) in DMSO from methacrylic acid and $Zr(OPr)_4$ or $ZrCl_4$ salts using X-ray determined clusters as structural reference. Free-energies are computed at 80ºC and 1M.

Entry	Reaction	ΔG	Ref.
1	**3**$Zr(OPr)_4$·2DMSO + 4McOOH → **Zr_3**$(O)(OOMc)_3(OPr)_7$ + 4PrOH + McOOPr + 6DMSO	-53.2	7
2	**3**$Zr(OPr)_4$·2DMSO + 6McOOH → **Zr_3**$(O)(OOMc)_5(OPr)_5$ + 6PrOH + McOOPr + 6DMSO	-64.6	7
3	**4**$Zr(OPr)_4$·2DMSO + 4McOOH → **Zr_4**$(O)_2(OOMc)_4(OPr)_{12}$ + 4PrOH + 8DMSO	-70.9	7
4	**4**$Zr(OPr)_4$·2DMSO + 4McOOH → **Zr_4**$(O)_2(OOMc)_2(OPr)_{10}$ + 4PrOH + 2McOOPr + 8DMSO	-61.0	7
5	**4**$Zr(OPr)_4$·2DMSO + 14McOOH → **Zr_4**$(O)_2(OOMc)_{12}$ + 14PrOH + 2McOOPr + 8DMSO	-109.1	8
6	**5**$Zr(OPr)_4$·2DMSO + 9McOOH → **Zr_5**$(O)_3(OOMc)_6(OPr)_8$ + 9PrOH + 3McOOPr + 10DMSO	-118.2	7
7	**6**$Zr(OPr)_4$·2DMSO + 20McOOH → **Zr_6**$(O)_4(OH)_4(OOMc)_{12}$ + 16PrOH + 8McOOPr + 12DMSO	**-201.3**	7
8	**3**$ZrCl_4$· + 8McOOH → **Zr_3**$(O)(OOMc)_7Cl_3$ + 8HCl + McOOCl	-6.7	7
9	**2**$ZrCl_4$· + 6McOOH → **Zr_2**$(OOMc)_4(HOOMc)_2Cl_4$ + 4HCl	-16.5	9
10	**6**$ZrCl_4$ + 20McOOH → **Zr_6**$(O)_4(OH)_4(OOMc)_{12}$ + 16HCl + 8McOOCl	**-19.7**	8

Table S4. Reaction free-energies (kcal mol^{-1}) for different decomposition pathways of the Zr_6 oxo cluster **12** in the presence of DMSO solvent and either water or propanol hydrolyzing agents. Free-energies are computed at 80 ºC and 1M.

Entry	Reaction	ΔG
1	**Zr_6**$(O)_4(OH)_4(OOMc)_{12}$ + H_2O + 3DMSO → **Zr_5**$(O)_3(OH)_4(OOMc)_{10}(DMSO)_2$ + **Zr**$(OH)_2(OOMc)_2(DMSO)$	+38.6
2	**Zr_6**$(O)_4(OH)_4(OOMc)_{12}$ + PrOH+ 3DMSO → **Zr_5**$(O)_3(OH)_4(OOMc)_{10}(DMSO)_2$ + **Zr**$(OH)(OOMc)_2(PrOH)(DMSO)$	+39.1
3	**Zr_6**$(O)_4(OH)_4(OOMc)_{12}$ +H_2O + 4DMSO → **Zr_4**$(O)_3(OH)_2(OOMc)_8(DMSO)_2$ + **Zr_2**$(OH)_4(OOMc)_4(DMSO)_2$	+53.0
4	**Zr_6**$(O)_4(OH)_4(OOMc)_{12}$ + 3DMSO → **Zr_4**$(O)_3(OH)_2(OOMc)_8(DMSO)_2$ + **Zr_2(O)**$(OH)_3(OOMc)_4(DMSO)$	+83.4
5	**Zr_6**$(O)_4(OH)_4(OOMc)_{12}$ + 6DMSO → 2**Zr_3**$(O)_2(OH)_2(OOMc)_6(DMSO)_3$	+56.4
6	**Zr_6**$(O)_4(OH)_4(OOMc)_{12}$ + 2DMSO + 4PrOH → 2**Zr_3**$(OH)_4(OOMc)_6(DMSO)(OPr)_2$	+79.4

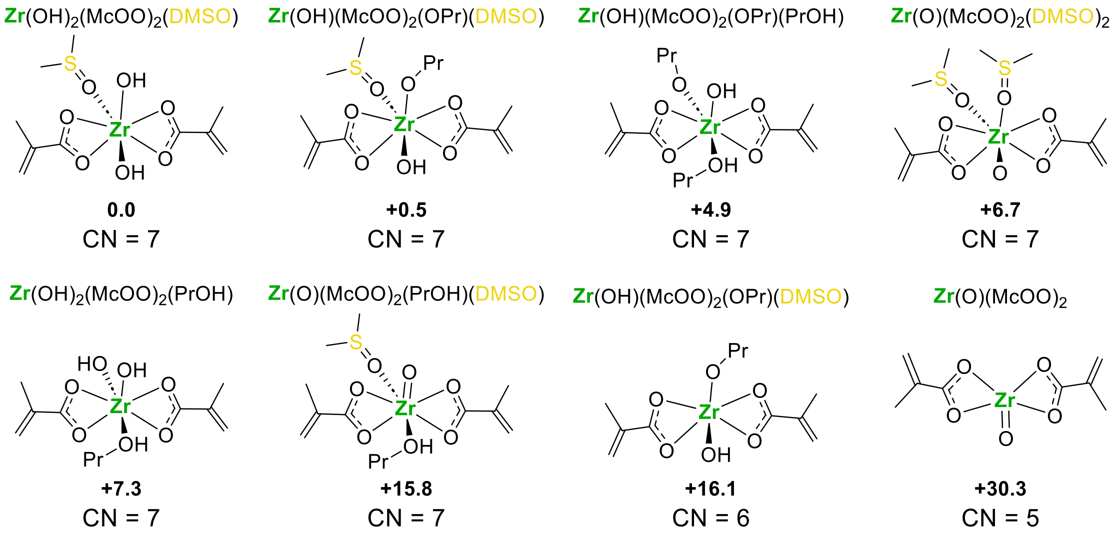

Figure S13. Exploration of possible monomeric Zr complexes under current experimental conditions, i.e., in the presence of H_2O, PrOH, McOH, and DMSO. Relative free energies in kcal·mol^{-1}, and Zr coordination numbers (CN) are given for each of the species.

Design of a suitable computational model

At the outset of our DFT study, we aimed to build a computational model of cluster **12** that would preserve computational efficiency, but accurately model the atomistic features of the catalytic site. Thus, we initially evaluated the thermodynamics of replacing a methacrylate capping ligand by a substrate molecule to identify the nature of the Zr centers that are most susceptible to ligand exchange. Ligand-exchange processes are a well-known feature of group (IV)-based metal oxo clusters,[5-6, 10] and our previous NMR experiments mixing **12** with substrate **11** indeed suggests ligand exchange as a facile process likely to initiate the amidation reaction.[11] Cluster **12** structure features three distinct coordination environments differing in the coordination mode of carboxylate ligands and local environment, and the energy differences among the three sites was found to span 4.1 kcal mol^{-1} only with lability increasing in the order 'chelating' < 'belt' < 'opposite' (**Figure 3b**). This is in line with the fast dynamics observed in previous experiments, and indicates 'opposite' sites as the most likely to be exchanged. However, participation of other sites or multiple exchanges cannot be excluded. Based on these results, we continued our study using a model in which the five methacrylate ligands not adjacent to the active site were replaced by formate linkers, and under the assumption that since both substrate and capping ligands are carboxylates, only the site where reaction is happening undergoes ligand exchange (**Figure S14**). In this way, we could still characterize the active site environment in detail, while keeping a good computational efficiency.

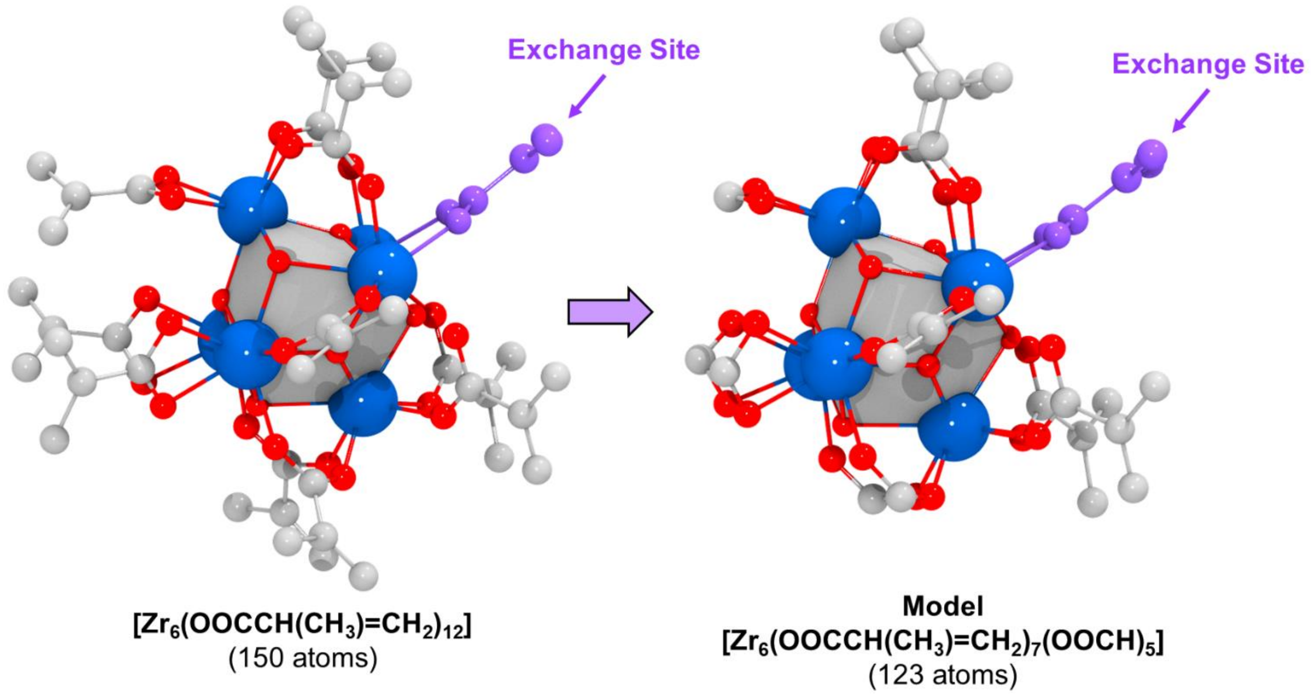

Figure S14. Pictorial representation of the reduction of the **12** model size used to improve the efficiency of the computational mechanistic investigation. Hydrogen atoms are omitted for clarity. Color code: Zr (light blue), O (red), C (gray).

Mechanistic investigation

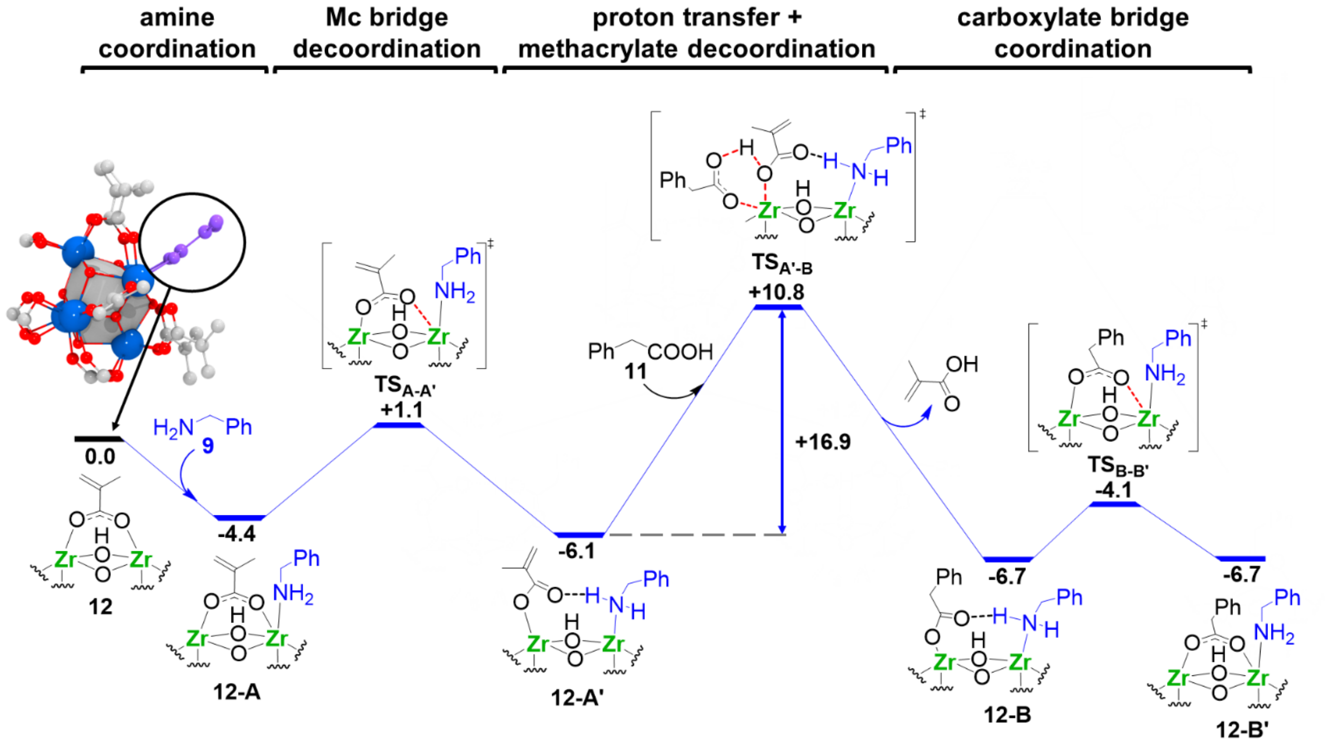

Figure S15. Gibbs free-energy profile (kcal mol^{-1}) for the ligand-exchange process of a methacrylate ligand by phenylacetic acid **11** through an associative, amine-assisted mechanism

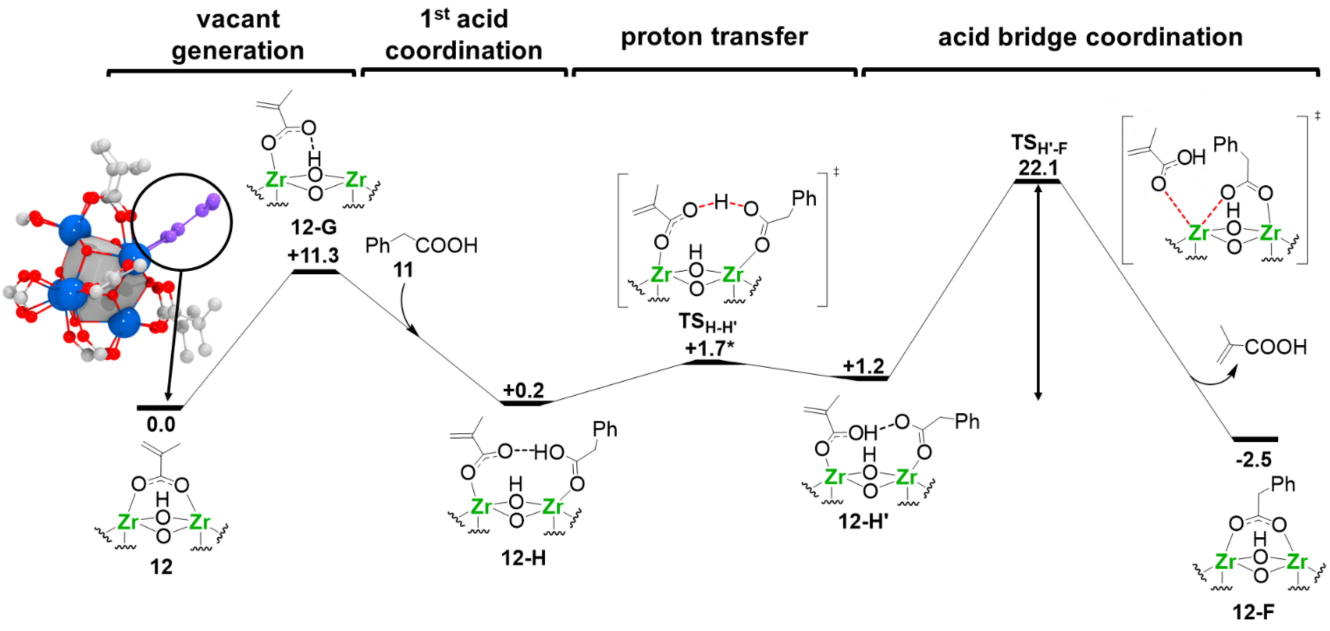

Figure S16. Gibbs free-energy profile (kcal mol^{-1}) for the ligand-exchange process of a methacrylate ligand by phenylacetic acid through a dissociative mechanism. This starts with the generation of a vacant site in one of the zirconium centers (**12-G**) as the ligand changes from bi- to monodentate, enabling the coordination of the acid substrate. This process has a free-energy cost of 11.3 kcal mol^{-1}. The determination of the corresponding transition state for dissociation was challenging, although we can assume a low barrier and that the free-energy difference a good estimated of the free-energy cost of this intramolecular dissociation. Once the phenyl-acetic acid occupies the empty Zr site (**12-H**), its proton is transferred to the non-coordinated methacrylate oxygen (**TS$_{H-H'}$**) obtaining the **12-H'** intermediate. The energy barrier (denoted with *) was estimated using 0 K electronic energies because, in this situation, thermal and entropic corrections shifted the free-energy below the product. The reaction proceeds through the release of the monodentate methacrylic acid from the Zr center, allowing the phenylacetic acid

to accommodate in a bidentate, bridging fashion ($\mathbf{TS_{H'-F}}$). This process requires overcoming an overall free-energy barrier of 22.1 kcal·mol^{-1}.

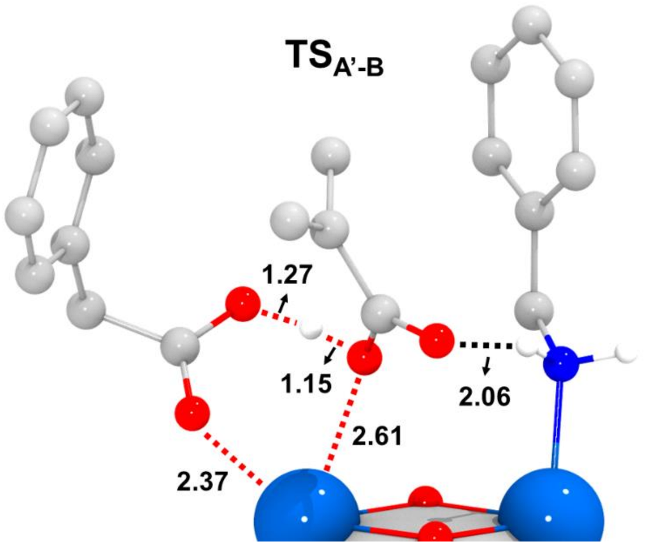

Figure S17. Balls-and-sticks representation of the transition-state structure for the ligand-exchange process ($\mathbf{TS_{A'-B}}$) involved in the associative, amine-assisted pathway shown in **Figure S1**. The most relevant distances are given in Å. Carbon-bound hydrogen atoms are omitted for clarity. Color code: Zr (light blue), O (red), C (gray), H (white), N (dark blue). Bonds being formed or broken in the transition-state geometry are highlighted with red dashed lines, and hydrogen bonds are shown in black dashed lines.

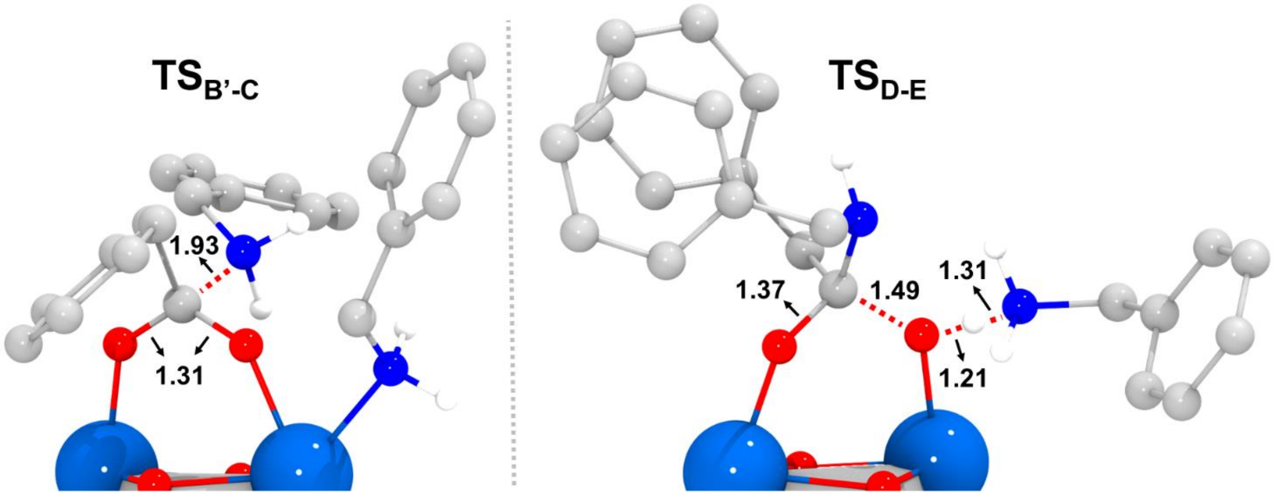

Figure S18. Balls-and-sticks representation of transition-state structures for the nucleophilic attack step responsible for the C—N bond formation ($\mathbf{TS_{B'-C}}$) and the second amine-mediated proton transfer step, which promote the scission of the C—O(H) bond ($\mathbf{TS_{D-E}}$) shown in Figure 3c. The most relevant distances are given in Å. Ligands and carbon-bound hydrogen atoms are omitted for clarity. Color code: Zr (light blue), O (red), C (gray), H (white), N (dark blue). Bonds being formed or broken in the transition-state geometry are highlighted with red dashed lines.

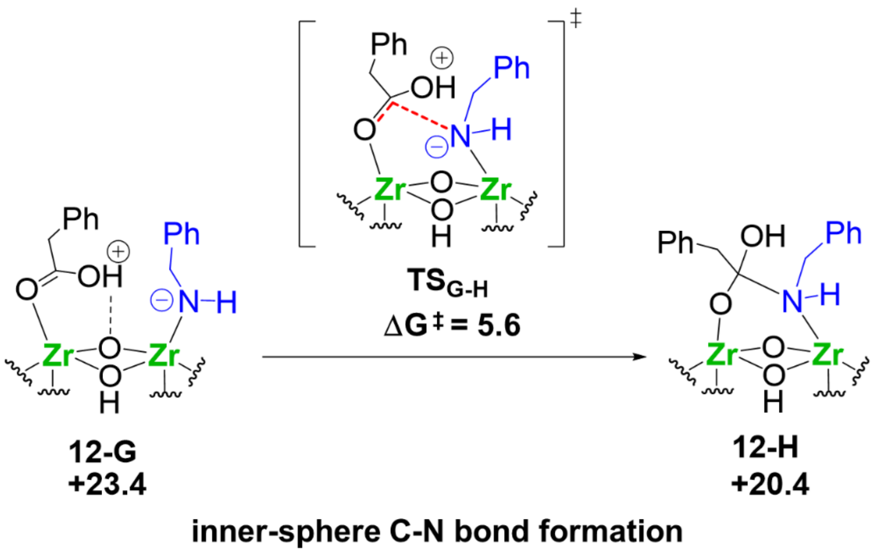

Figure S19. Representation of the unfeasible inner-sphere C-N bond formation. The unstable intermediate **12-G** would be originated through a N,O proton transfer from **12-B'** which would move the carboxylic acid from bi- to mono-coordinated as it gets protonated. The process goes through a nucleophilic attack from the amido group towards the carboxylic acid giving intermediate **12-H** in a Gibbs free-energy barrier of 5.6 kcal·mol^{-1}. All relative free-energies are referred to the starting species **12** in kcal·mol^{-1}.

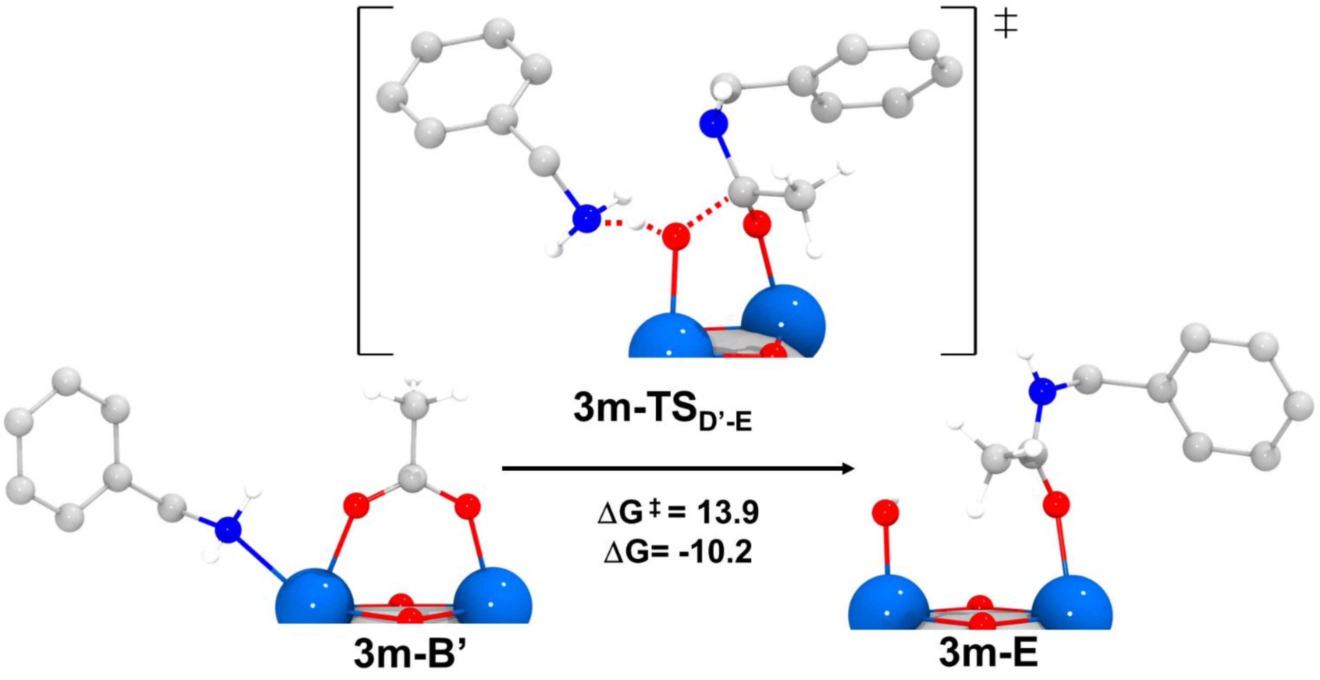

Figure S20. Balls-and-sticks representation of the most relevant calculated structures for the amide bond formation between acetic acid (**2**) and benzylamine (**9**) catalyzed by the *in situ* formed Zr cluster **3** which was computed as a monomeric model (**3m**). The intermediate **3m-B'** was found as the resting state, the transition step **3m-TS$_{D'-E}$** as the rate determining step where there is the second amine-mediated proton transfer step while the C—O(H) bond is cleaved and **3m-E** was found as the product. The given free energies are in kcal·mol^{-1}. Color code: Zr (light blue), O (red), C (gray), H (white), N (dark blue). Bonds being formed or broken in the transition-state geometry are highlighted with red dashed lines.

Mechanism of amide bond formation catalyzed by mononuclear Zr species

Figure S21 depicts the computed mechanism for the peptide bond formation by mononuclear Zr salt ($Zr(Mc)(BnNH_2)Cl_3$) that would be originated from $ZrCl_4$. The process starts by a nucleophilic attack of an external amine through a barrierless process. Subsequently, the protonated nitrogen atom, transfers the proton to one of the carbonyl oxygens through a 2-step process where an external amine behaves as a proton shuttle. After proton transfer, the C−O(H) bond cleavage takes place and constitutes the rate-determining step.

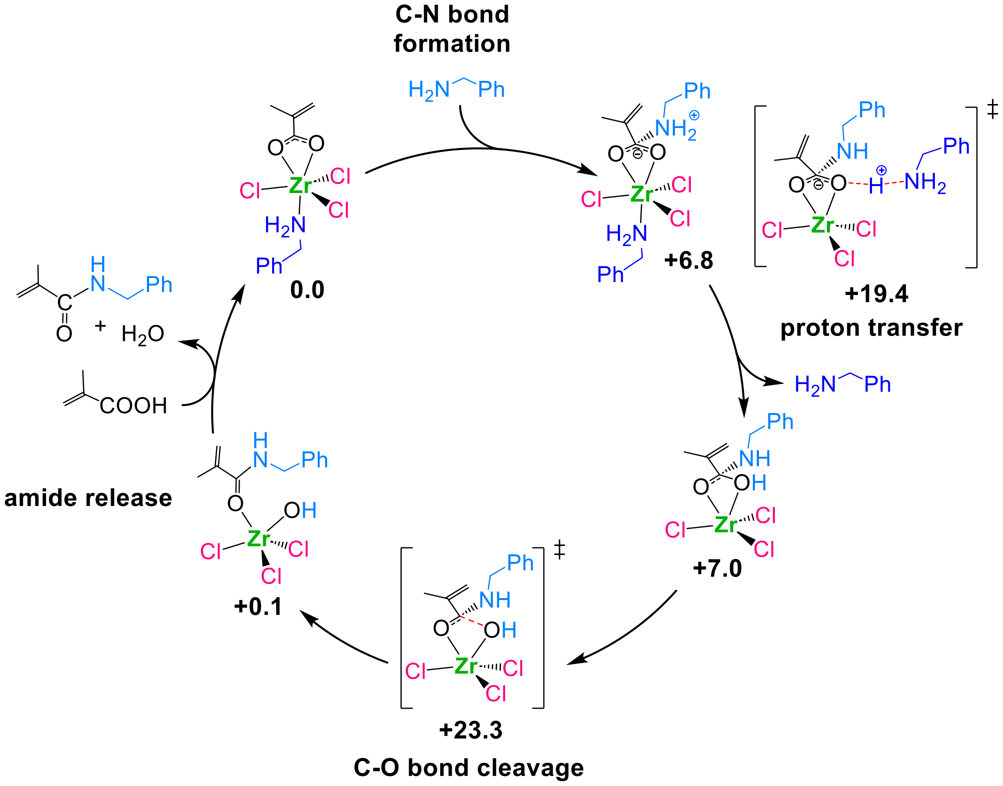

Figure S21. Catalytic cycle for the amide bond formation process between a methacrylate ligand and benzylamine catalyzed by a $ZrCl_4$ salt. Gibbs free-energies in kcal·mol^{-1}.

Molecular Dynamics study of ZrOCs clusters in solution: water and DMSO solvent mixtures

We carried out atomistic Molecular Dynamics (MD) simulations with explicit solvent molecules to investigate the interactions of Zr clusters with the solvent and other species involved in the reaction; aiming to rationalize experimental observation indicating that this catalyst does not require water scavenger to enable amide formation reactions. For these simulations, we selected the hexazirconium cluster with methacrylate capping ligands, **12**. All simulated systems include one molecule of **12** embedded in a 3D-periodic DMSO solvent box, in the presence of additional species at distinct proportions: i) 7 H_2O molecules, initially, interacting, through hydrogen-bonding, with the coordination sphere of Zr cluster (**Figure S22** and **Figure S23**) ii) 1:10 mixture of water and DMSO, in whose initial configuration water is solvating the Zr cluster (**Figure S24**); iii) 7:7:3:23 mixture of water, amide product (**14**), and reactants phenylacetic acid (**11**), and benzylamine (**9**) in excess, mimicking reaction conditions with a 70% of yield relative to the initial amount of benzylic acid (**Figure S25**).

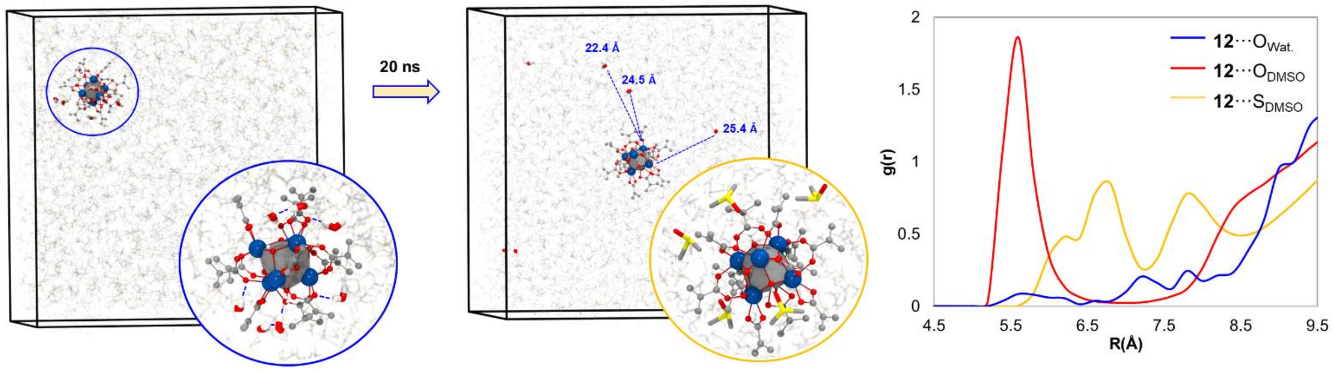

Figure S22. From left to right: (1) Initial configuration of the 3D-periodic cubic simulation box (63^3 Å^3) for the hexazirconium cluster **12** in DMSO with 7 H_2O molecules interacting through hydrogen-bonds with the carboxylate ligands and the protonated Zr-bridged μ_3-OH groups. The inset shows a closer look at the interactions of water with **12**; (2) configuration after 20 ns of simulation at 343 K (closer look at the Zr cluster first solvation sphere, where water molecules are not present); and (3) Radial Distribution Function (RDF) between the center of mass of the Zr cluster and the water oxygen (blue line), DMSO oxygen (red line), and DMSO sulfur (yellow line) atoms averaged over the last 10 ns of the simulation. All RDFs converge into 1 at ca. 30 Å. The peak at 5.8 Å in the **12**···O_{DMSO} RDF (red line) integrates to 3 oxygens; the peaks in the **12**···S_{DMSO} RDF (yellow line) integrate to 4 sulfur atoms at a distance cutoff of 7 Å (after the first broad peak), and to 11 at a distance cutoff of 8.5 Å; and the **12**···$O_{Wat.}$ RDF (blue line) integrates to ca. 0.1 at 9.5 Å. H atoms of DMSO are omitted for clarity.

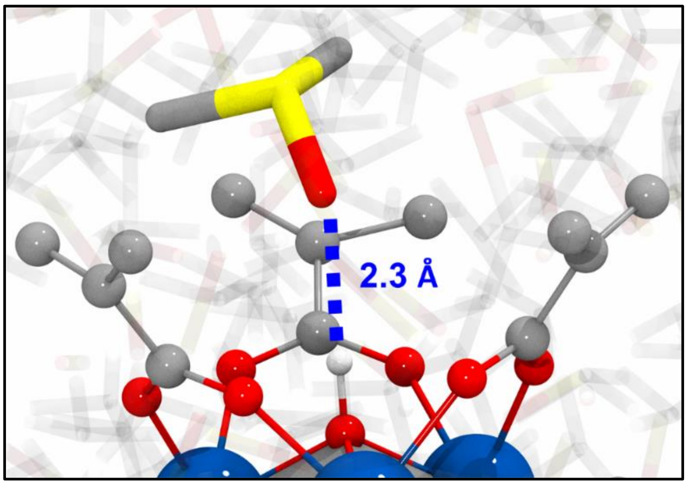

Figure S23. Representative snapshot of the interaction between the Zr cluster and the solvent extracted from the trajectory of **12** in DMSO with 7 H_2O molecules. These interactions chiefly involve hydrogen bonds between the oxygen of DMSO and the hydrogen of μ_3-OH groups of the ZrOC (average S=O···H(O) distance of 2.3 Å). All RDFs converge into 1 at ca. 30 Å. H atoms of DMSO are omitted for clarity.

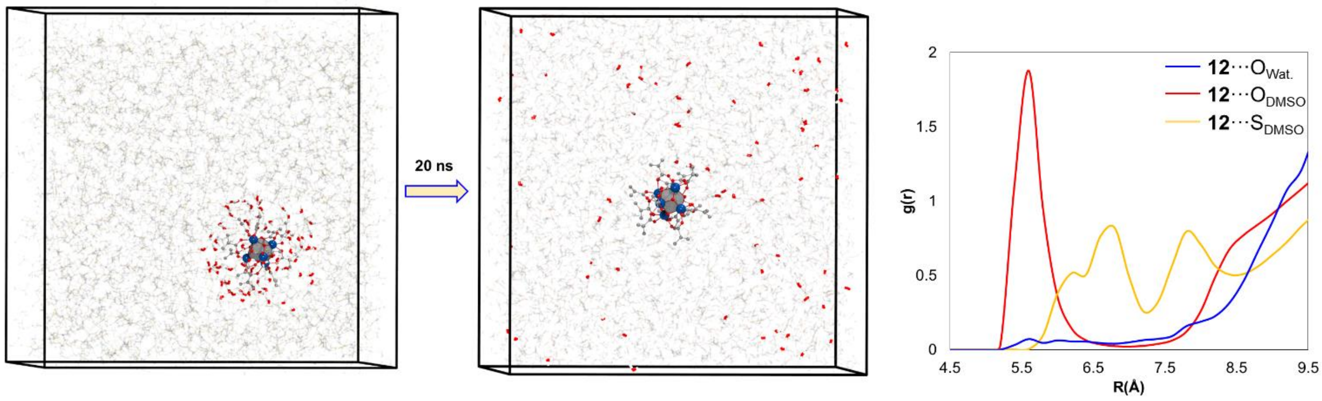

Figure S24. From left to right: (1) Initial configuration of the 3D-periodic cubic simulation box (63^3 $Å^3$) for hexazirconium cluster (**12**) in DMSO with 10% v/v of H_2O (73 molecules) placed around the Zr cluster **12**; (2) configuration after 20 ns of simulation at 343 K; and (3) Radial Distribution Function (RDF) between the center of mass of the Zr cluster and the water oxygen (blue line), DMSO oxygen (red line), and DMSO sulfur (yellow line) atoms averaged over the last 10 ns of the simulation. All RDFs converge into 1 at ca. 30 Å. The peak at 5.8 Å in the **12**···O_{DMSO} RDF (red line) integrates to 3 oxygens atoms; the peaks in the **12**···S_{DMSO} RDF (yellow line) integrate to 4 at a distance cutoff of 7 Å (after the first broad peak); and to 11 at a distance cutoff of 8.5 Å; and the **12**···O_{Wat} (blue line) integrates to ca. 1 at 9.5 Å.

Firstly, we generated an initial configuration in which 7 H_2O molecules are directly interacting with the first coordination sphere of Zr cluster **12**, forming hydrogen bonds with the oxygens of carboxyl ligands and the hydrogen of Zr-bridged μ_3-hydroxo group (see **Figure S22**-1). After few ns of simulation at 343 K, we observed that interacting water molecules leave the solvation sphere of Zr cluster and diffuse into the DMSO bulk (**Figure S22**-2). Accordantly, the RDF of water molecules around the ZrOC does not show any unambiguous peak, indication the absence of a preferred contact distance (**Figure S22**-3, blue line). On the other hand, the RDF of oxygen atoms of DMSO around the ZrOC shows a relatively sharp peak centred at ~5.6 Å that integrates to approximately 3 DMSO molecules, which correspond to DMSO molecules interacting with the Zr cluster through hydrogen bonds between the DMSO oxygen and the μ_3-OH groups of the cluster as illustrated in the snapshot of **Figure S23**. The RDF peaks between sulphur DMSO and the Zr cluster are broader and with less defined shape and corresponds to the first solvation shell of DMSO around **12** (**Figure S22-3**, yellow line). Increasing the water content surrounding the ZrOC, from 7 to 72 molecules (approximately 10% in volume), we observed the same behaviour as it is shown in **Figure S24**. Overall, these simulations indicate that Zr cluster **12** has a marked hydrophobic character caused by the organic substituent of the carboxylates decorating the cluster. Thus, water molecules coming from wet solvents or produced during the formation of amides are expelled from the coordination sphere of the Zr-cluster by hydrophobic forces. This could have a positive effect on the reaction by preventing the reverse reaction, the peptide hydrolysis, explaining why water scavengers are not experimentally required for amide bond formation promoted by ZrOCs.

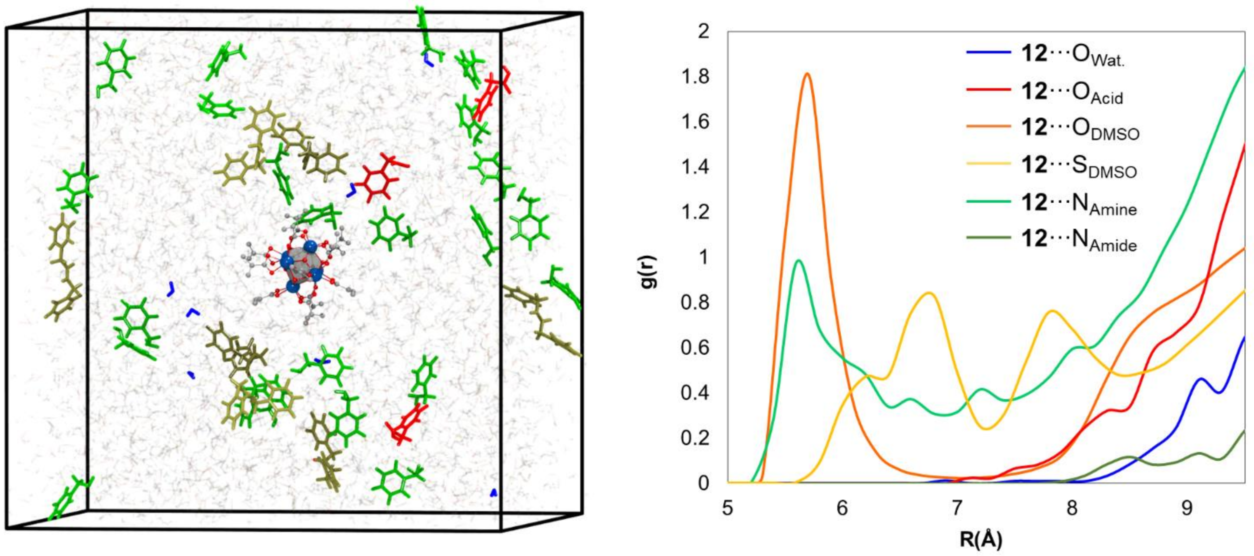

Figure S25. From left to right: (1) final configuration of the simulation at 373 K of hexazirconium cluster **12** in DMSO box of 63^3 Å^3, and in the presence of 7 molecules of water (dark blue), 7 molecules of amide product **14** (dark green)¸3 molecules of phenylacetic acid **11** (red), and benzylamine reactant **9** (light green), mimicking the proportion obtained at the end of the reaction; (2) Radial Distribution Function (RDF) between the center of mass of the Zr cluster and the water oxygen (blue line), the O(H) of the acid (red line), the DMSO oxygen (orange line), and DMSO sulfur (yellow line), the nitrogen of the amine reactant **11** (ligh green line), and nitrogen of amide product **14** (dark green line), averaged over the last 10 ns of the simulation. All RDFs converge into 1 at ca. 30 Å. The peak at 5.8 Å in the **12**···O_{DMSO} RDF (orange line) integrates to 3 oxygen atoms; the **12**···S_{DMSO} RDF (yellow line) integrates to 4 at a distance cutoff of 7 Å (after the first broad peak), and to 11 at a distance cutoff of 8.5 Å; the **12**···$O_{Wat.}$ RDF (blue line) integrates to ca. 0.1 at 9.5 Å; the **12**···N_{Amine} RDF (light green line) integrates to 0.05 at 6.5 Å; and the **12**···N_{Amide} and **12**···O_{Acid} RDFs (dark green and red lines, respectively) integrate to 0.5 and 0.02 at 9.5 Å, respectively.

Finally, we carried out a simulation of Zr cluster **12** in DMSO, including other species present in the reaction such as water, benzylamine **9**, phenylacetic acid **11**¸and amide product **14**. To better mimic experimental conditions the ratio of these species assumed the 70% of the observed conversion, the 3-fold excess of the amine, resulting in the following mixture ratio, 7:7:3:23 for water:**14**:**11**:**9**. **Figure S25** collects the results of this simulation. The interaction of DMSO and water with the Zr-cluster show the same features as previous simulations: (1) no preferred interaction with water molecules; and (2) a solvation shell of DMSO molecules, interacting preferentially through the oxygen atom via formation of weak hydrogen bonds with the μ_3-OH groups of the Zr cluster. Neither the acid nor the amide molecules show a preferred interaction with the cluster in the RDF plot (**Figure S25**-2). For amines, which are present in larger proportion, the RDF plot displays a small peak centred at ~5.6 Å, which is the same distance as that for RDF peak of DMSO oxygen (**Figure S25**-2, light blue line), suggesting that the excess of amine competes with DMSO in the solvation of the Zr cluster, and that the directionality of the interaction through the nitrogen atom could be related to the observed amine coordination.

References

1. Puchberger, M.; Kogler, F. R.; Jupa, M.; Gross, S.; Fric, H.; Kickelbick, G.; Schubert, U. Can the Clusters $Zr_6O_4(OH)_4(OOCR)_{12}$ and $[Zr_6O_4(OH)_4(OOCR)_{12}]_2$ Be Converted into Each Other? *Eur. J. Inorg. Chem.* **2006**, *2006*, 3283-3293. https://doi.org/10.1002/ejic.200600348

2. Van den Eynden, D.; Pokratath, R.; Mathew, J. P.; Goossens, E.; De Buysser, K.; De Roo, J. Fatty acid capped, metal oxo clusters as the smallest conceivable nanocrystal prototypes. *Chem. Sci.* **2023**, *14*, 573-585. http://dx.doi.org/10.1039/D2SC05037D

3. Dai, S.; Simms, C.; Dovgaliuk, I.; Patriarche, G.; Tissot, A.; Parac-Vogt, T. N.; Serre, C. Monodispersed MOF-808 Nanocrystals Synthesized via a Scalable Room-Temperature Approach for Efficient Heterogeneous Peptide Bond Hydrolysis. *Chem. Mater.* **2021**, *33*, 7057-7066. https://doi.org/10.1021/acs.chemmater.1c02174

4. Filipponi, A.; Di Cicco, A. X-ray-absorption spectroscopy and n-body distribution functions in condensed matter. II. Data analysis and applications. *Phys. Rev. B* **1995**, *52*, 15135-15149. https://link.aps.org/doi/10.1103/PhysRevB.52.15135

5. Walther, P.; Puchberger, M.; Kogler, F. R.; Schwarz, K.; Schubert, U. Ligand dynamics on the surface of zirconium oxo clusters. *Physical Chemistry Chemical Physics* **2009**, *11*, 3640-3647. http://dx.doi.org/10.1039/B820731C

6. Chiu, C.-C.; Shieh, F.-K.; Tsai, H.-H. G. Ligand Exchange in the Synthesis of Metal–Organic Frameworks Occurs Through Acid-Catalyzed Associative Substitution. *Inorg. Chem.* **2019**, *58*, 14457-14466. https://doi.org/10.1021/acs.inorgchem.9b01947

7. Boyle, T. J.; Ottley, L. A. M.; Rodriguez, M. A. Structurally characterized carboxylic acid modified zirconium alkoxides for the production of zirconium oxide thin films. *Polyhedron* **2005**, *24*, 1727-1738. http://www.sciencedirect.com/science/article/pii/S0277538705003724

8. Kickelbick, G.; Schubert, U. Oxozirconium Methacrylate Clusters: $Zr_6(OH)_4O_4(OMc)$ and $Zr_4O_2(OMc)_{12}$ (OMc = Methacrylate). *Chem. Ber.* **1997**, *130*, 473-478. https://doi.org/10.1002/cber.19971300406

9. Lundberg, H.; Tinnis, F.; Zhang, J.; Algarra, A. G.; Himo, F.; Adolfsson, H. Mechanistic Elucidation of Zirconium-Catalyzed Direct Amidation. *J. Am. Chem. Soc.* **2017**, *139*, 2286-2295. https://doi.org/10.1021/jacs.6b10973

10. Van den Eynden, D.; Pokratath, R.; De Roo, J. Nonaqueous Chemistry of Group 4 Oxo Clusters and Colloidal Metal Oxide Nanocrystals. *Chem. Rev.* **2022**, *122*, 10538-10572. https://doi.org/10.1021/acs.chemrev.1c01008

11. Zhang, Y.; Kokculer, I. Y.; de Azambuja, F.; Parac-Vogt, T. N. Dynamic Environment at the Zr_6 Oxo Cluster Surface Is Key for the Catalytic Formation of Amide Bonds. *Catal. Sci. Technol.* **2023**, *13*, 100-110. http://dx.doi.org/10.1039/D2CY01706G
